# Challenges and opportunities for managing aquatic mercury pollution in altered landscapes

**DOI:** 10.1007/s13280-017-1006-7

**Published:** 2018-01-31

**Authors:** Heileen Hsu-Kim, Chris S. Eckley, Dario Achá, Xinbin Feng, Cynthia C. Gilmour, Sofi Jonsson, Carl P. J. Mitchell

**Affiliations:** 1 Department of Civil & Environmental Engineering, Duke University, 121 Hudson Hall, Box 90287, Durham, NC 27708 USA; 2 U.S. Environmental Protection Agency, Region-10, 1200 6th Ave, Seattle, WA 98101 USA; 3 Unidad de Calidad Ambiental, Instituto de Ecología, Carrera de Biología, Universidad Mayor de San Andrés, P.O. Box 10077, La Paz, Bolivia; 4 State Key Laboratory of Environmental Geochemistry, Institute of Geochemistry, Chinese Academy of Sciences, Guiyang, 550002 China; 5 Smithsonian Environmental Research Center, 647 Contees Wharf Rd, Edgewater, MD 21037-0028 USA; 6 Department of Environmental Science and Analytical Chemistry, Stockholm University, Svante Arrhenius väg 8, 11418 Stockholm, Sweden; 7 Department of Physical and Environmental Sciences, University of Toronto Scarborough, 1265 Military Trail, Toronto, ON M1C 1A4 Canada

**Keywords:** Contamination, Landcover, Mercury synthesis, Methylmercury

## Abstract

The environmental cycling of mercury (Hg) can be affected by natural and anthropogenic perturbations. Of particular concern is how these disruptions increase mobilization of Hg from sites and alter the formation of monomethylmercury (MeHg), a bioaccumulative form of Hg for humans and wildlife. The scientific community has made significant advances in recent years in understanding the processes contributing to the risk of MeHg in the environment. The objective of this paper is to synthesize the scientific understanding of how Hg cycling in the aquatic environment is influenced by landscape perturbations at the local scale, perturbations that include watershed loadings, deforestation, reservoir and wetland creation, rice production, urbanization, mining and industrial point source pollution, and remediation. We focus on the major challenges associated with each type of alteration, as well as management opportunities that could lessen both MeHg levels in biota and exposure to humans. For example, our understanding of approximate response times to changes in Hg inputs from various sources or landscape alterations could lead to policies that prioritize the avoidance of certain activities in the most vulnerable systems and sequestration of Hg in deep soil and sediment pools. The remediation of Hg pollution from historical mining and other industries is shifting towards in situ technologies that could be less disruptive and less costly than conventional approaches. Contemporary artisanal gold mining has well-documented impacts with respect to Hg; however, significant social and political challenges remain in implementing effective policies to minimize Hg use. Much remains to be learned as we strive towards the meaningful application of our understanding for stakeholders, including communities living near Hg-polluted sites, environmental policy makers, and scientists and engineers tasked with developing watershed management solutions. Site-specific assessments of MeHg exposure risk will require new methods to predict the impacts of anthropogenic perturbations and an understanding of the complexity of Hg cycling at the local scale.

**Electronic supplementary material:**

The online version of this article (10.1007/s13280-017-1006-7) contains supplementary material, which is available to authorized users.

## Introduction

Global efforts sparked by the Minamata Convention are underway to reduce releases of mercury (Hg) to the environment (Selin et al. 2018). These efforts, in addition to global perturbations such as climate change, have the potential to greatly alter the worldwide distribution and impact of Hg, as described in companion papers (Eagles-Smith et al. 2018; Obrist et al. 2018; Selin et al. 2018). Hg exposure risk can be ameliorated by strategic management of individual ecosystems. In this paper we review and evaluate the many site-specific human activities and alterations to landscapes that can affect Hg transport, methylation, and bioaccumulation, including mining, forestry operations, urbanization, rice cultivation, nutrient loadings, wetland and reservoir creation and management, and industrial contamination.

**Fig. 1:**
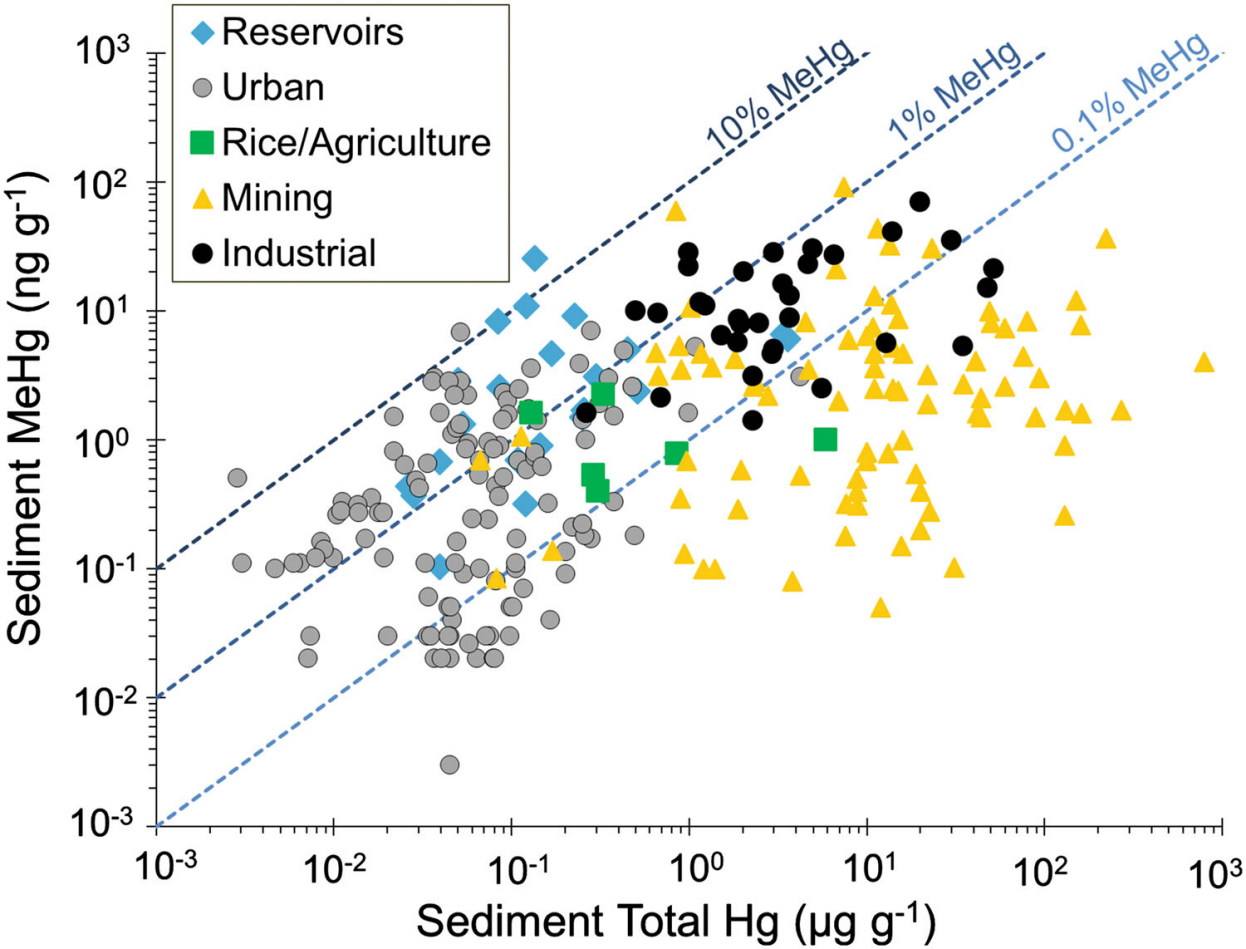
Total Hg and MeHg contents observed at more than 200 aquatic sites that have been perturbed by anthropogenic activities. The risks of Hg exposure at these sites generally depend on the mobilization potential of Hg from the site as well as the potential for MeHg bioaccumulation and exposure to wildlife and humans. References for the data used in the figure are provided in the supplementary material

While Hg released into the environment is typically in an inorganic form, concerns about human and wildlife exposure are mostly related to monomethylmercury (MeHg) that accumulates in fish and other food. MeHg is produced in ecosystems through naturally occurring processes that convert inorganic Hg to MeHg. These processes and the extent of MeHg bioaccumulation are key aspects of the Hg cycle and depend on a number of site-specific conditions. To illustrate the critical role of ecosystem processes in Hg exposure risk, Fig. 1 shows the broad range of MeHg levels found in the sediments and soils of more than 200 perturbed systems, spanning a wide range of Hg contamination. MeHg content as a fraction of total Hg spans three to four orders of magnitude at any given total Hg content.

To evaluate the impacts of natural and anthropogenic perturbations on Hg exposure risk, we must consider the complex array of atmospheric, hydrological, biological, ecological, and geochemical processes that control Hg transport and transformation to MeHg in the environment, as described in many previous reviews (Ullrich et al. 2001; Selin 2009; Liu et al. 2012b; Lucotte et al. 2012; Driscoll et al. 2013; Hsu-Kim et al. 2013) and highlighted briefly here. Atmospheric Hg is primarily in the gaseous elemental form Hg^0^, which has a relatively long atmospheric lifetime allowing for its widespread distribution (Schroeder and Munthe 1998). The oxidation of Hg^0^ in the atmosphere is a key process that governs the spatial distribution of wet and dry Hg deposition to land or water (Selin 2009). Deposited Hg can be highly reactive towards further transformation such as photochemical reduction (Amyot et al. 1997; Schroeder and Munthe 1998), incorporation into vegetation (Graydon et al. 2012; Rea et al. 2002), chelation to dissolved natural organic matter (NOM) (Aiken et al. 1998, 2011), and sorption to particles (e.g., organic matter, minerals, microorganisms) (Gerbig et al. 2012; Liu et al. 2012c; Skyllberg 2012; Vost et al. 2012). In addition to direct atmospheric deposition, Hg release into waterways can originate from upland runoff, industrial and mining point sources, and remobilization and resuspension of contaminated sediment and soil (Selin 2009; Driscoll et al. 2013). Hg released to waterways is generally strongly chelated (e.g., Hg-NOM) or associated with particles (both particulate NOM and mineral particles) (Han and Gill 2005; Hsu-Kim and Sedlak 2005; Balogh et al. 2008; Schuster et al. 2008; Dittman et al. 2010). Thus, factors that influence NOM and particle mobilization are critical drivers of Hg transport.

**Fig. 2:**
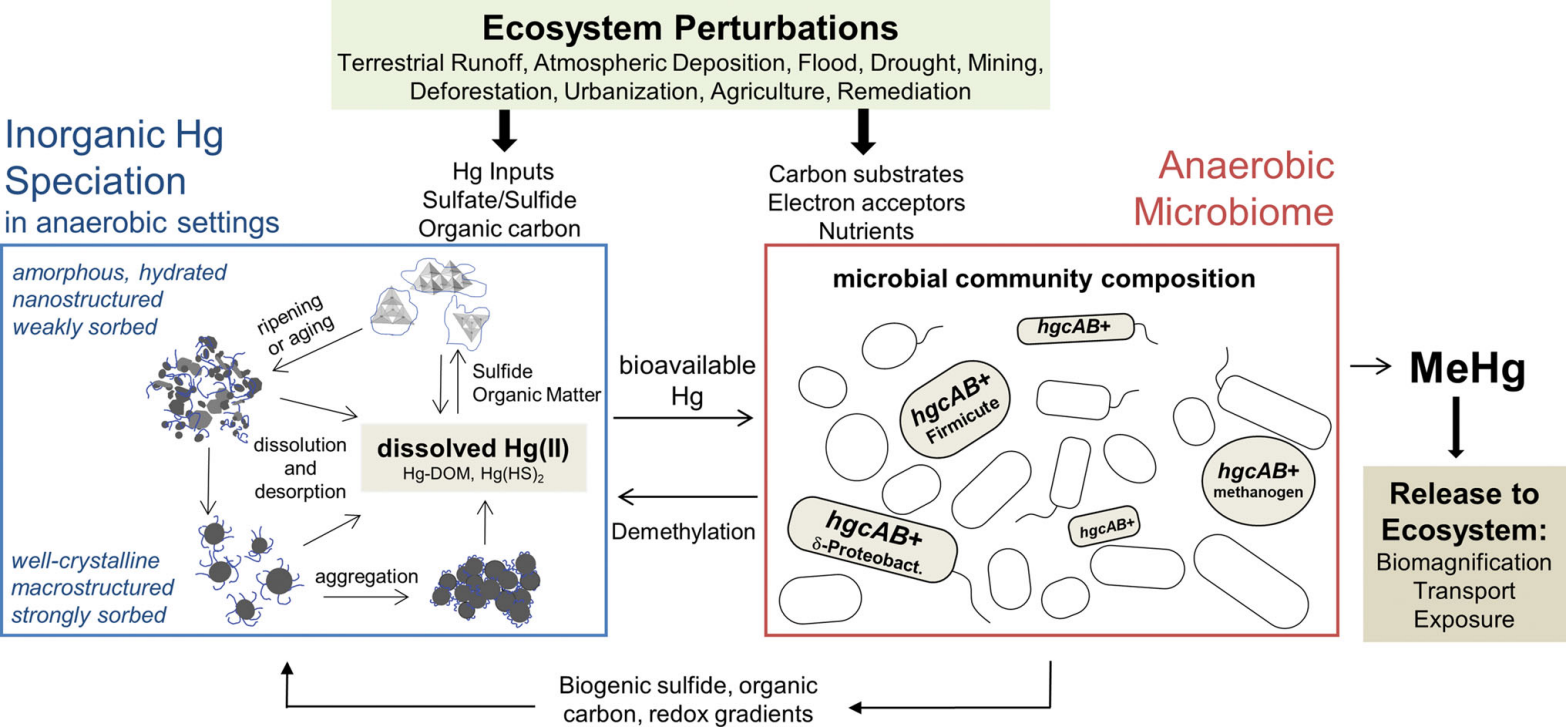
Perturbations to ecosystems may affect key factors that contribute to the production of MeHg in the aquatic environment. These factors include the geochemical speciation (bioavailability) of inorganic Hg, the productivity of methylating microorganisms, and the degradation of MeHg. In most anaerobic environments, inorganic Hg is predominantly associated with particles comprising sulfides and natural organic matter (NOM). The relative bioavailability of particulate Hg can vary greatly between ‘newer’ forms (e.g., weakly sorbed, amorphous, or nanostructured species) compared to ‘older’ aging states (e.g., strongly sorbed, well-crystalline, macrostructured species). Hg methylation rates also depend on the growth and productivity of *hgcAB*+ microorganisms, which entail a wide diversity of species that can be roughly grouped into three major clades: δ-Proteobacteria, Firmicutes, and methanogens. This anaerobic microbiome will also alter the chemical composition of its environment (e.g., sulfide, organic carbon, redox potential) that can subsequently alter Hg speciation and bioavailability

Recently deposited, transported, or mineralized Hg tends to be more reactive towards methylation and bioaccumulation than “old” Hg that has aged in place in sediments and soil (Hintelmann et al. 2002; Paterson et al. 2006; Harris et al. 2007; Orihel et al. 2008; Jonsson et al. 2014, 2017). The aging effect for Hg may stem from the relative differences in bioavailability of Hg forms; weakly sorbed, amorphous, or nanostructured Hg forms may be more soluble at the bacterial cell envelope compared to more recalcitrant forms of Hg (e.g., strongly sorbed to particulate matter, sparingly soluble microcrystalline Hg mineral forms) (Fig. 2) (Deonarine and Hsu-Kim 2009; Graham et al. 2012; Jonsson et al. 2012; Zhang et al. 2012b; Pham et al. 2014). Knowledge of the relative bioavailability of inorganic Hg species could allow site managers to develop strategies that distinguish exposure risks between multiple sources of Hg. However, we still lack tools that can quantitatively classify the sources of Hg with respect to methylation and food web accumulation potential. The challenge is due to the complexity of the Hg biogeochemical cycle in aquatic ecosystems as well as limitations of existing measurement capabilities.

The conversion of inorganic Hg to MeHg in the environment is primarily a microbially driven process that is commonly present in anaerobic sediments, saturated soils, anoxic bottom waters, as well as anoxic engineered systems (e.g., wastewater treatment, bioreactors) (Barkay and Wagner-Dobler 2005). There is also evidence of MeHg production in O_2_-containing surface oceans (Lehnherr et al. 2011; Lamborg et al. 2014; Gionfriddo et al. 2016), although the mechanisms of this process are not well understood. Hg-methylating microorganisms identified to date include sulfate reducers, iron reducers, methanogens, and a handful of fermentative and syntrophic Firmicutes (Compeau and Bartha 1985; Fleming et al. 2006; Kerin et al. 2006; Ranchou-Peyruse et al. 2009; Gilmour et al. 2013a; Yu et al. 2013; Podar et al. 2015). The diversity of these organisms is still being realized; however, they all share the *hgcA* and *hgcB* gene cluster that encodes for proteins involved in intracellular methylation of inorganic Hg(II) (Parks et al. 2013). Methylating organisms are prevalent in benthic aquatic settings [e.g., saturated soil and sediment (Gilmour et al. 1992; Branfireun et al. 1999; King et al. 2000, 2002; Hines et al. 2006; Monperrus et al. 2007; Mitchell and Gilmour 2008; Avramescu et al. 2011)] as well other microenvironments with steep redox gradients (e.g., periphyton, biofilms, microbial flocs) (Mauro et al. 2001; Achá et al. 2011; Yu et al. 2013; Hamelin et al. 2015; Ortiz et al. 2015; Podar et al. 2015; Gascón Díez et al. 2016; Olsen et al. 2016).

MeHg can also be degraded by biotic and abiotic processes. These include photochemical decomposition pathways (Sellers et al. 1996; Hammerschmidt and Fitzgerald 2006; Lehnherr and St. Louis 2009; Hammerschmidt and Fitzgerald 2010; Zhang and Hsu-Kim 2010; Black et al. 2012) and the microbial Hg detoxification pathway encoded by the *mer* operon, which can both demethylate MeHg and reduce inorganic Hg(II) to Hg^0^ (Barkay and Wagner-Dobler 2005). The *mer* system is found mainly in aerobic bacteria and is believed to be inducible with sufficient Hg exposure to the organism. MeHg degradation in anaerobic niches can be rapid (Hines et al. 2012; Tjerngren et al. 2012; Cesário et al. 2017), but much less is known about microbial MeHg decomposition and dark abiotic demethylation processes (e.g., via sulfides) in anaerobic settings (Craig and Moreton 1984; Oremland et al. 1991; Wallschlager et al. 1995; Jonsson et al. 2016).

While establishing the net production of MeHg in aquatic systems would be a major step towards understanding the potential risk of Hg pollution, the bioaccumulation and biomagnification of MeHg in the food web determines mercury exposure to humans and wildlife. The process of MeHg bioaccumulation depends on many ecological factors, though food web dynamics appears to be the primary driver of MeHg levels in fish and other wildlife, rather than microbial methylation of Hg in benthic environments (Eagles-Smith et al. 2018).

Overall, many factors influence Hg mobilization, transformation, and food web accumulation. While this complexity can be daunting for environmental managers and regulators to address, it also means that there are multiple approaches that could be pursued to reduce MeHg levels in biota. In the following text, we consider a variety of ecosystem-scale stresses and perturbations, their effects on Hg cycling in aquatic systems, and possible opportunities for policy and research to mitigate negative consequences.

## Altered surface loadings

**Fig. 3:**
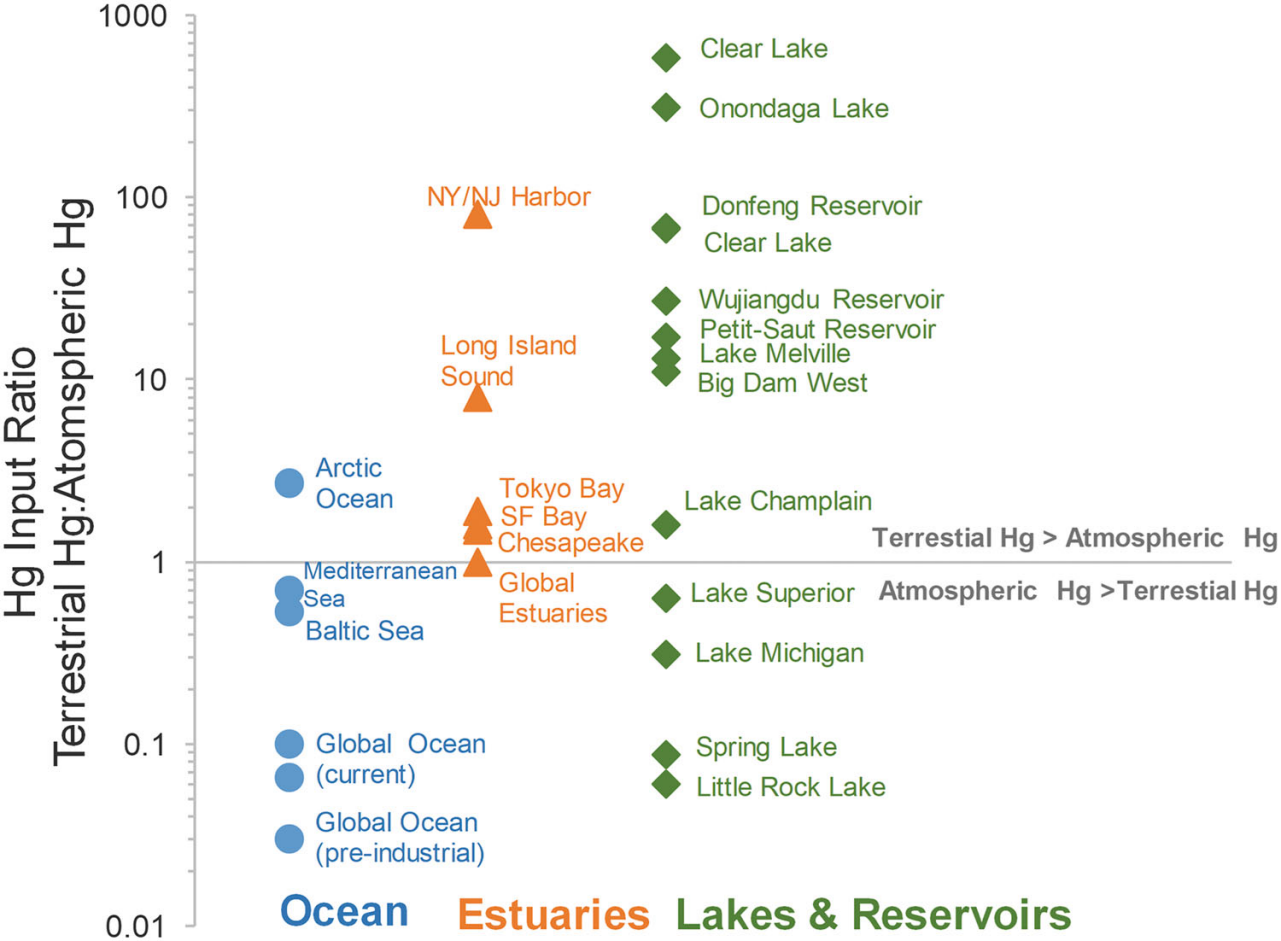
Ratio of estimated Hg mass inputs from terrestrial sources (including surface and subsurface hydrological fluxes) relative to direct atmospheric Hg deposition to surface water for a variety of aquatic systems. Ecosystems with large terrestrial Hg:atmospheric Hg input ratios are expected to respond more slowly to changes in global Hg emissions relative to ecosystems with low ratios that are projected to respond more quickly. References for data shown in the figure are provided in Table S1

Controls on global atmospheric Hg emissions and subsequent reduction of Hg loadings to surface waters are expected to result in an eventual decrease of Hg bioaccumulation in fisheries. The timing of this response, however, has substantial uncertainty due to the legacy of stored Hg in terrestrial and sediment compartments of watersheds and the variability in Hg retention times among watersheds (Munthe et al. 2007). For example, Hg directly deposited to surface waters from the atmosphere has been observed to methylate and bioaccumulate in aquatic food webs relatively quickly (e.g., within months to a year), while Hg deposited to upland terrain generally requires much more time (a decade or more) for subsequent impact on pelagic food webs (Harris et al. 2007; Oswald et al. 2014). Because of this time difference, the response of individual water bodies to increases or decreases in Hg loadings is expected to vary widely with the relative contributions of atmospheric and terrestrial Hg inputs (Fig. 3, Table S1). Direct atmospheric deposition of Hg tends to dominate over runoff inputs in marine water bodies. In contrast, lakes and estuaries tend to have greater terrestrial Hg inputs relative to atmospheric Hg deposition. This ratio depends on surface water area relative to watershed drainage area, the type of land cover within the drainage area (e.g., forested, urban, etc.), and the presence of known historical point sources of Hg.

While upland soils in watersheds are known to be long-term sources of total inorganic Hg to downstream surface waters, they can also be major sources of MeHg due to methylation of Hg and storage of MeHg in saturated soils (e.g., forest and wetland areas) (St. Louis et al. 1994; Mitchell et al. 2008a, 2009; Chen et al. 2012). Land use changes that impact flow paths, including forestry practices as described below, can exacerbate MeHg flux from watersheds (e.g., Shanley et al. 2008; Flanders et al. 2010; Babiarz et al. 2012). This increase in MeHg flux from upland soils directly to surface waters could provide a direct path towards bioaccumulation of MeHg in the pelagic food web, especially compared to other potential sources of MeHg such as sediments that would enter the water column at a slower rate (Jonsson et al. 2014). Terrestrial runoff of NOM can also change the base of the food web from autotrophic to heterotrophic production, resulting in a shift of the food web structure and subsequent MeHg bioaccumulation pathways (Jonsson et al. 2017).

The loading rates of other key constituents (e.g., organic carbon, sulfur, nitrogen, phosphorous) can also alter the biogeochemical cycle of Hg. For example, organic carbon loading to aquatic ecosystems impacts Hg cycling in a variety of ways. Dissolved and particulate NOM tends to be the vehicle of Hg transport in surface waters. Thus, increased organic carbon loads often result in increased Hg loads (Schuster et al. 2008, 2011; Brigham et al. 2009; Scudder 2009). Organic matter production or release can contribute to eutrophication and increase the extent and duration of anoxic conditions in thermally stratified water bodies. These changes affect redox gradients which can result in increased MeHg production (Driscoll et al. 1995; Slotton et al. 1995; Watras et al. 1995; Herrin et al. 1998; Eckley and Hintelmann 2006; Merritt and Amirbahman 2008). Chelation of inorganic Hg(II) by NOM has the potential to lower the bioavailability of Hg for methylation; however, this effect might be masked by stimulation of Hg methylators, depending on the type of NOM (Drott et al. 2007; Kim et al. 2011; Gascón Díez et al. 2016; Mazrui et al. 2016; Bravo et al. 2017) and the limiting factor for net Hg methylation (e.g., Hg speciation, productivity of methylators, or demethylation processes) (Jonsson et al. 2012; Zhang et al. 2014b; Kucharzyk et al. 2015; Liem-Nguyen et al. 2016). Organic matter loads also influence the structure of pelagic food webs, with secondary impacts on MeHg biomagnification (Jonsson et al. 2017). Taken together, these processes illustrate how organic carbon cycling in watersheds is intertwined with Hg cycling in complex and non-linear relationships.

The impact of sulfate loadings on Hg cycling is well documented. Sulfate originating from atmospheric deposition, upland runoff, and industrial sources can stimulate the activity of sulfate-reducing bacteria in peatlands and freshwater ecosystems, resulting in enhanced MeHg production rates (Gilmour et al. 1992; Branfireun et al. 1999; Jeremiason et al. 2006; Mitchell et al. 2008b; Coleman Wasik et al. 2012; Akerblom et al. 2013). Thus, reduction of sulfurous acid deposition has the potential to also reduce MeHg production and bioaccumulation (Hrabik and Watras 2002; Watras and Morrison 2008; Coleman Wasik et al. 2012). While this impact is straightforward, a secondary effect of elevated sulfate loading is enhanced microbial production of sulfide (e.g., Bailey et al. 2017). Inorganic sulfide in turn alters the distribution of dissolved and particulate Hg in benthic settings (Fig. 2), depending on the relative amounts of Hg, sulfide, and organic matter in the system. The development of models that can successfully discern the speciation of Hg (or even the bioavailable fraction) in environmental samples remains a major unmet need within research, management, and policy communities.

Increased loadings of nutrients (nitrogen and phosphorous) to lakes and coastal systems and subsequent eutrophication can alter Hg biogeochemistry in numerous ways. For example, eutrophication can lower the concentrations of MeHg in biota via biodilution and growth dilution, a process that lowers concentrations of MeHg in primary producers and consumers due to increased biomass (Pickhardt et al. 2002; Chen and Folt 2005; Pickhardt et al. 2005; Kim et al. 2008; Luengen and Flegal 2009; Gosnell et al. 2017). In addition, eutrophication can alter organic carbon loads to surface waters, resulting in mixed effects that can increase or decrease MeHg levels in surface waters, as noted above. Managed alterations such as additions of nitrate or hypolimnetic oxygenation in the field have been attempted with some success to change redox conditions and decrease MeHg concentrations in water, but these manipulations do not always result in reductions of MeHg in biota (Matthews et al. 2013; Austin et al. 2016; Beutel et al. 2016; McCord et al. 2016).

Due to the complex nature of the biogeochemical cycle of Hg, predicting the net effect of altered loadings of Hg, MeHg, organic carbon, nutrients, sulfate, and other constituents on MeHg accumulation in organisms remains a significant challenge. In this respect, management solutions that utilize a watershed loadings approach (such as total maximum daily loads in the U.S.) are difficult to formulate. While much remains to be learned, conceptual models could still be useful for understanding the main effects of altered surface loadings. For example, predictions on the impacts of eutrophication have been successful in some cases (e.g., wastewater nutrient inputs) but not others (e.g., estuarine nutrient export to coastal waters) (Driscoll et al. 2012). Regardless, these models highlight the relative importance of eutrophication, including biodilution, increased sedimentation of Hg, and the potential for increased in situ production of MeHg (Driscoll et al. 2012; Soerensen et al. 2016). Likewise, identification of the chemical forms (or aging states) of Hg and MeHg that enter surface waters can help guide policies that prioritize reductions of certain sources, or perhaps assign value terms to individual sources as a basis for economic incentives or trading programs for discharge permits. These management approaches will require a richer understanding of uncertainties associated with the assignment of values, and in particular, methods to quantify Hg and MeHg bioavailability to relevant organisms.

## Forestry and deforestation

Undisturbed forested ecosystems can effectively sequester Hg via accumulation in vegetation and soils (Obrist et al. 2011). As a result, forested ecosystems typically have relatively low runoff yields, averaging 6 ± 2 % of atmospheric Hg deposition (Mason et al. 1997; Balogh et al. 2008; Shanley et al. 2008; Domagalski et al. 2016). In streams and rivers draining from harvested catchments, increases in total Hg and MeHg concentrations have been observed in many (but not all) studies and have been attributed to increased soil erosion, Hg methylation activity, and/or mobilization of near surface Hg pools in soils (Roulet et al. 1999; Fostier et al. 2000; Roulet et al. 2000; Porvari et al. 2003; Mainville et al. 2006; Allan et al. 2009; Sorensen et al. 2009; Lacerda et al. 2012; Eklof et al. 2013, 2014; de Wit et al. 2014; Kronberg et al. 2016a; Ukonmaanaho et al. 2016). Soil erosion can occur as a result of vegetation removal, road construction, and/or other forest harvesting practices. In addition to erosion, the burning of forest slash following logging (as often occurs in tropical regions where forests are converted to agriculture) has been shown to increase Hg mobilization via inundation of the otherwise poor soils with cations (Farella et al. 2006; Beliveau et al. 2009; Comte et al. 2013).

**Fig. 4:**
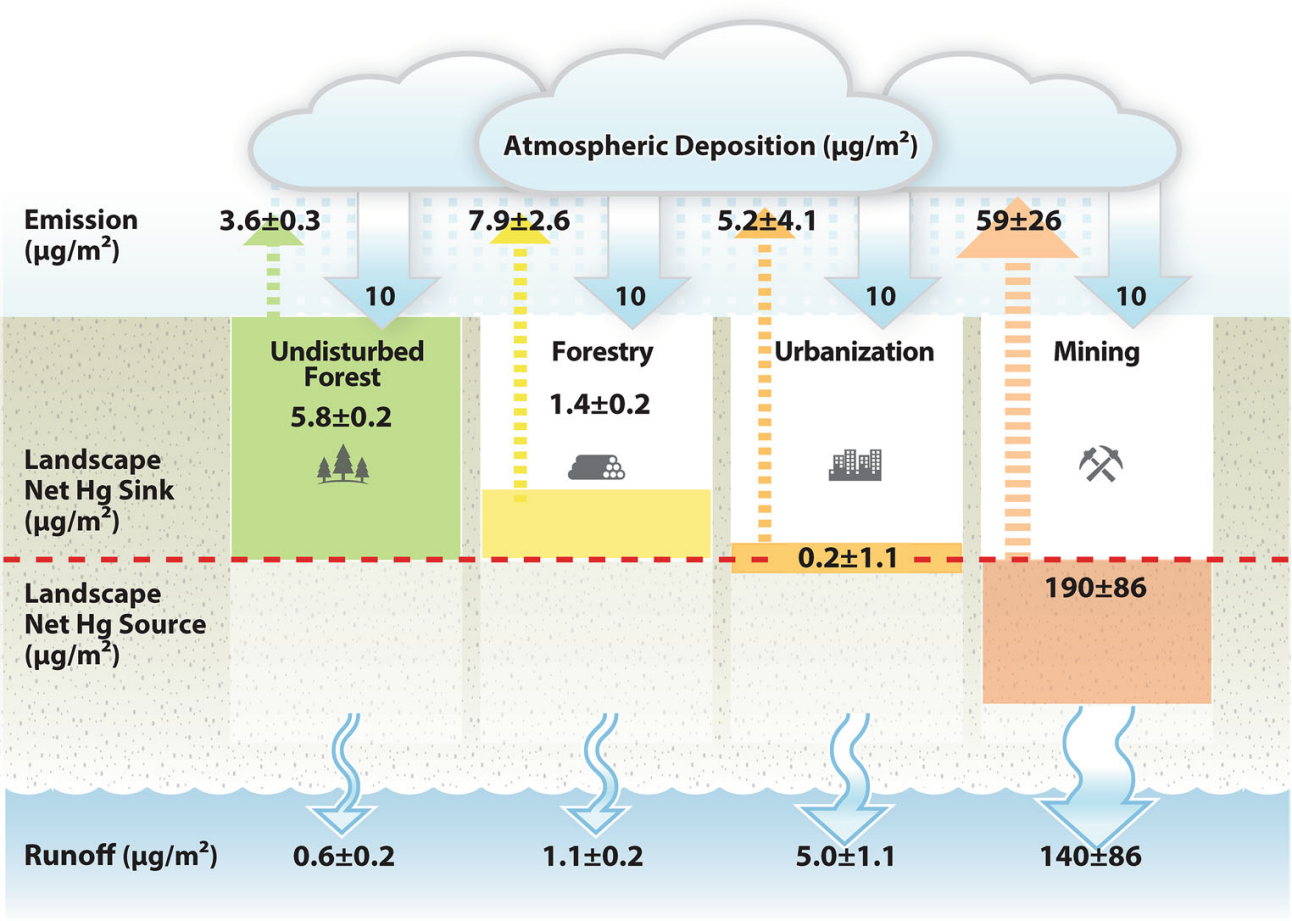
The influence of different landscape perturbations on Hg accumulation within catchments and Hg exports via runoff and emission. Mean (±SE) accumulation and export loads were obtained from field studies of undisturbed forest, forested, urbanized, and mine-impacted catchments and have been scaled relative to a constant atmospheric deposition (10 μg/m^2^). The calculations and references used to create this figure are available in the supplementary material

Regardless of the impact of forestry operations on Hg concentrations, the fluxes of Hg from harvested catchments are typically two-fold higher than that from undisturbed forests, largely due to increases in stream discharge following logging (Porvari et al. 2003; Allan et al. 2009; Sorensen et al. 2009; de Wit et al. 2014; Eklof et al. 2014; Kronberg et al. 2016b) (Fig. 4). Discharge can increase due to reductions in evapotranspiration, interception, and infiltration as well as modified snow accumulation and melt rates. Forest harvesting can also increase MeHg production and/or mobilization through several mechanisms. For example, decreased evapotranspiration can lead to an elevated water table, increased soil moisture, and ponding, all of which foster anoxic conditions favorable for methylation (Munthe and Hultberg 2004; Braaten and de Wit 2016; Kronberg et al. 2016a). Furthermore, fresh organic carbon inputs from logging debris left on the site may enhance microbial activity and MeHg production (Eklof et al. 2016; Kronberg et al. 2016a). However, increased MeHg concentrations in streamwater draining harvested watersheds have only been clearly observed in some studies (Porvari et al. 2003; Skyllberg et al. 2009; Eklof et al. 2012), whereas others have shown no significant change in MeHg response to forestry operations (Allan et al. 2009; Eklof et al. 2013; de Wit et al. 2014; Kronberg et al. 2016a). The differing responses among studies, for MeHg as well as total Hg, are most likely due to site-specific variations in harvesting practices (e.g., the degree of soil disturbance), catchment characteristics (e.g., water table depth, slope, hydrological flow paths), and meteorological differences (e.g., timing and amount of precipitation). As such, it remains difficult to make broad generalizations about the impacts of forestry operations on Hg cycling and MeHg production.

Greater solar radiative fluxes reach the soil in harvested catchments, leading to warmer temperatures, which facilitate photoreduction and evasion of soil-bound Hg (Mazur et al. 2014). In addition, deposition of gaseous Hg to vegetation can have a large impact on the net amount of Hg released from a landscape, and the reduction in plant uptake following forestry operations increases the net evasion of Hg to the atmosphere (Eckley et al. 2016). Releases of Hg from the surface following forest harvesting may be similar or larger in magnitude to losses via aqueous fluxes (Mazur et al. 2014; Gamby et al. 2015; Eckley et al. 2016). Despite increased releases to air and water, harvested catchments are still expected to be a net-sink for atmospheric Hg inputs, albeit less efficient ones compared to undisturbed forests (Fig. 4).

In addition to catchment-scale impacts, several studies in boreal and temperate forests have shown that logging activity correlates with increased Hg concentrations in sediment and aquatic biota in downstream waterbodies (Garcia and Carignan 1999, 2000; Sampaio Da Silva et al. 2005; Desrosiers et al. 2006; Van Furl et al. 2010). The degree to which forestry operations contribute to the variability of Hg concentrations in fish among lakes may be relatively minor compared to other factors such as physiography and climate, and the impact is less detectable in larger lakes (Lucotte et al. 2016). An understudied area is how the potential impacts of forest harvesting on other biogeochemical constituents, particularly nutrients, alters the overall ecology of downstream aquatic ecosystems. This knowledge gap obscures the impacts on Hg bioaccumulation in receiving waters through processes such as growth dilution and biodilution (as noted in the previous Sect. 2). Augmented NOM transport from watersheds as a function of harvesting, in addition to being a potential vector for Hg transport, may also impact photochemical transformations and thus the pools of inorganic Hg (O’Driscoll et al. 2004) and MeHg (Klapstein et al. 2017) in lake waters.

Harvesting and site preparation methods that minimize machinery damage of soils (e.g., winter harvesting on frozen ground) and promote rapid revegetation appear to result in little to no downstream MeHg impact (Sorensen et al. 2009), whereas practices such as stump harvesting, mounding, and scarification can lead to significant or highly variable impacts depending on site characteristics (Munthe and Hultberg 2004; Eklof et al. 2012, 2014). Other forestry best management practices, such as the protection of streamside management zones and riparian buffers, generally offer improved water quality outcomes (Lakel et al. 2010), but there is a limited understanding of how these practices influence total Hg and MeHg cycling. Other common management practices, such as the use of well-designed, located, and maintained log landings, skid trails, and forest roads (Brown et al. 2015), have not been well studied in relation to the management of Hg mobility and contamination, but one could postulate that surface erosion control is a strategy that can decrease downstream Hg transport in nearly all instances.

In summary, the impact of forestry operations and deforestation on Hg is variable among catchments and forestry practices, but generally involves alterations to watershed loadings of Hg, organic carbon, and nutrients. Much research remains to be conducted in ecosystems outside of boreal forests of the northern latitudes, particularly in tropical regions. Regardless, relatively well-established best management practices involving careful site selection and protections against soil disturbance and erosion should help alleviate the magnitude and impacts of Hg transport and transformation.

## Reservoir creation

**Fig. 5:**
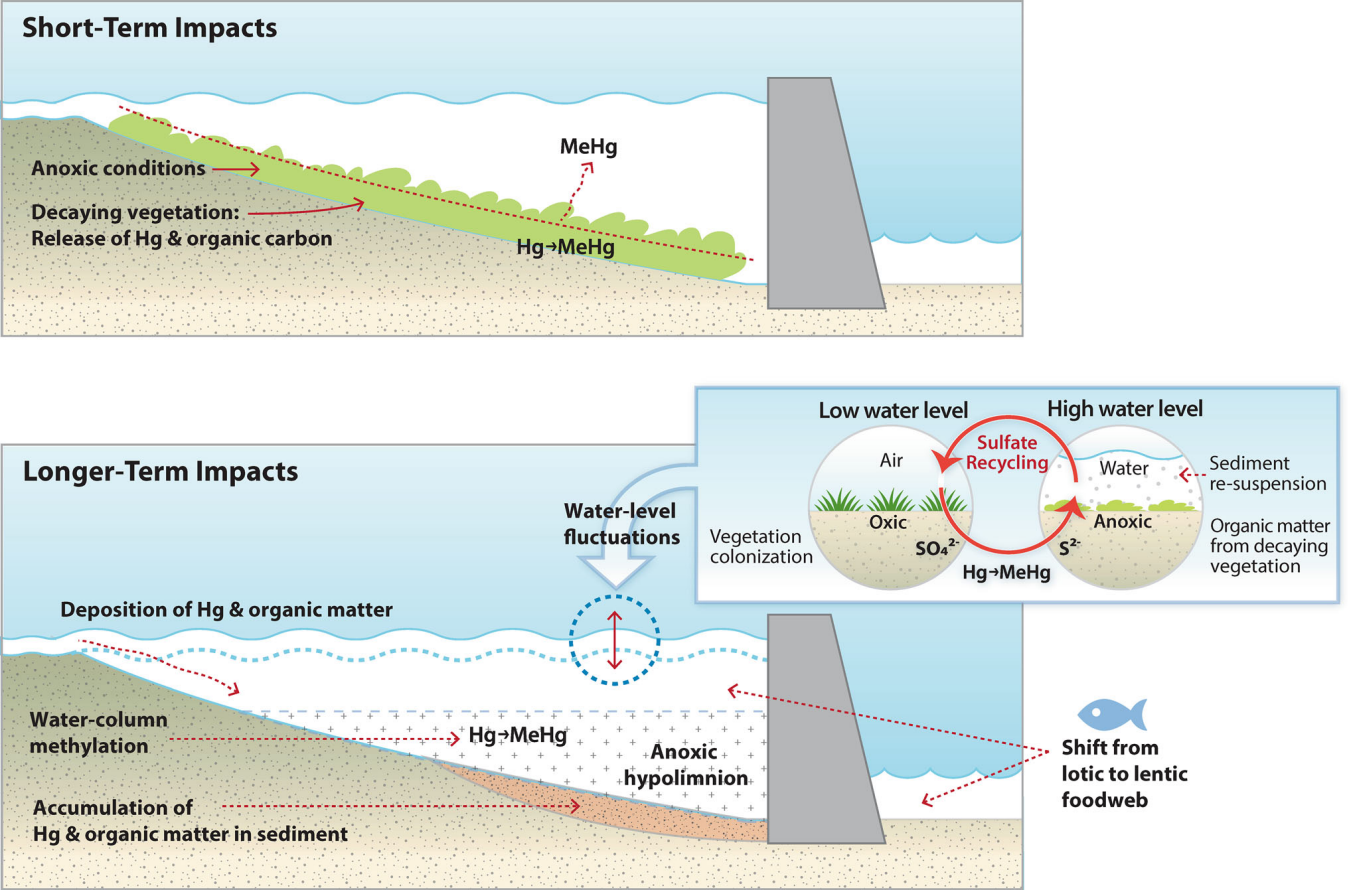
Conceptual diagram showing the short-term and longer-term impacts of reservoir creation on MeHg cycling and bioaccumulation. The short-term impacts of increased MeHg production are highly dependent on the organic matter content of the flooded catchment, with some reservoirs projects located in low organic matter watersheds showing no increase in MeHg

The impoundment of rivers and streams and the subsequent creation of reservoirs are among the most common anthropogenic manipulations of freshwater aquatic ecosystems. Reservoirs typically aim to provide flood control or water supplies for irrigation and electricity utilities. Their impacts on Hg bioaccumulation have been closely studied in numerous locations. Globally, the total area impounded by dams may rival the total surface area of natural lakes (St. Louis et al. 2000) and continues to grow, particularly in tropical regions of the southern hemisphere (Shiklomanov and Rodda 2004; Tuomola et al. 2008). The impacts of reservoir creation on Hg cycling typically result in an increase in MeHg production and can occur as part of shorter-term impacts following the construction of the reservoir as well as ongoing impacts due to hydrological, ecological and biogeochemical changes (Fig. 5).

For newly created reservoirs, the increase in MeHg production and bioaccumulation results from the decomposition of flooded terrestrial organic material, which leads to increased microbial activity and increased net MeHg production in flooded soils (Mucci et al. 1995; Porvari and Verta 1995; Kelly et al. 1997; Hall and Louis 2004; Hall et al. 2004; St. Louis et al. 2004; Hall et al. 2009). However, there are several studies from reservoirs in China where elevated fish Hg concentrations did not follow impoundment (Horvat et al. 2003; He et al. 2008; Feng et al. 2009; Liu et al. 2012a; Li et al. 2015a), likely due to low catchment organic matter content. Based on this observation, reductions in catchment organic matter prior to flooding have been proposed as a means to mitigate Hg impacts before reservoir creation (Mailman and Bodaly 2006).

Research over the last four decades has demonstrated that reservoirs can have elevated fish Hg concentrations compared to rivers and natural lakes (Meister et al. 1979; Montgomery et al. 2000; Brigham et al. 2002; Kamman et al. 2005); however, the extent and timing of effects varies considerably. In large piscivorous fish in boreal Canadian reservoirs, Hg levels have been shown to increase three- to six-fold after flooding and remain above pre-impoundment levels for several decades (Bodaly et al. 2004, 2007; Schetagne and Therrien 2013). The extent of MeHg bioaccumulation at these sites was high enough to possibly impact fish reproduction and growth (Scheuhammer et al. 2007), although fish growth and yield may improve after reservoir formation in oligotrophic systems (Bilodeau et al. 2015). For Quebec reservoirs, peak MeHg levels occurred at 4–9 years for non-piscivorous fish and 9–11 years for piscivorous fish (Bilodeau et al. 2015). For hundreds of western US and Canadian reservoirs, the average peak in fish Hg occurred only 3 years after flooding, and rarely exceeded 3-fold of background (Willacker et al. 2016). Lower organic carbon stores (soil and vegetation) in western catchments relative to boreal areas may be a driver of these differences in fish Hg.

In addition to increased MeHg production following the initial flooding, the ongoing wetting and drying cycles, as well as reservoir drawdown, can continue to affect MeHg production in older reservoirs (Orem et al. 2011; Eckley et al. 2015). During dry periods, reduced sulfur and organic matter stored in anaerobic soils can oxidize, fueling a pulse of MeHg production on each rewetting cycle. Additionally, sulfide production during wet periods modifies dissolved NOM with reduced sulfur moieties that enhance Hg availability for methylation (Graham et al. 2012; Poulin et al. 2017). Sediment wetting and drying cycles can increase the breakdown of organic matter, which can result in increased partitioning of sediment-bound Hg into the porewater phase and increased dissolved organic carbon production, both of which have been shown to enhance Hg methylation in reservoirs (Eckley et al. 2017). Finally, reservoir water-level fluctuations have also been shown to increase sediment erosion and resuspension of Hg in the water column, which may make it more available for methylation (Mucci et al. 1995).

As noted above, observations of reservoirs in China have suggested less impact (relative to North American sites) on fish Hg concentrations (Larssen 2010). In addition to the relatively low organic matter content, the reservoirs in China had higher flow rates, shorter food webs, and biodilution and growth dilution that may contribute to lower fish Hg concentrations (Horvat et al. 2003; He et al. 2008; Feng et al. 2009; Liu et al. 2012a; Li et al. 2015a). However, unlike the research on reservoirs from North America, in which a decrease in fish Hg concentrations is predicted as the reservoir ages, reservoirs in China may experience an increase in MeHg production over time as sediment organic matter accumulates from both allochthonous and autochthonous sources (Feng et al. 2009). Autochthonous organic matter can increase as nutrients are trapped within the reservoir and the stagnant water conditions created by the reservoir increase the production of algal biomass.

Tools to predict the timing and magnitude of reservoir creation on MeHg accumulation in food webs can help guide resource management decisions (Calder et al. 2016). Early comparisons among Canadian and Finnish reservoirs showed that reservoir age, size, temperature, and organic matter content predicted the magnitude of the reservoir effect on MeHg bioaccumulation (Rudd et al. 1983; Verta et al. 1986; Bodaly et al. 1993). Simple regression models using percent of total reservoir area flooded, or the ratio of flooded area:volume were shown to predict 75–85 % of variability in fish Hg for northern Canadian reservoirs (Johnston et al. 1991; Bodaly et al. 2007), especially if the upstream flooded area was included in the model. However, we still lack models that can reliably predict responses over a wide range of conditions.

The available data and models are heavily biased to boreal and temperate systems even though most current reservoir construction projects (and some of the largest reservoirs in the world) are located in the tropics and sub-tropics. Notably, boreal Canadian reservoirs may be a worst-case scenario for organic matter decomposition after flooding, due at least in part to large carbon stores in peat (St. Louis et al. 2000). Furthermore, model predictions of the impacts of reservoir creation can be complicated, even in the well-studied boreal forest region of Canada. For example, regression models of existing reservoirs suggest that the upper limit on peak increases in fish Hg within reservoirs is about six-fold above pre-impoundment levels (Johnston et al. 1991; Bodaly et al. 2007). Another model that used a probabilistic approach incorporating a large variety of aquatic foods, site-specific bioaccumulation factors, and an explicit prediction of the impacts downstream of the reservoir predicted at 10-fold increase (Calder et al. 2016).

There are several options to minimize MeHg production during reservoir construction and ongoing management. Locations with Hg-contaminated soils or areas receiving high loads of Hg in runoff or via atmospheric deposition should be avoided as new reservoir construction sites. Multiple reservoirs constructed in series may compound MeHg accumulation (Feng et al. 2009). The harvesting of vegetation before flooding and extension of fill times could reduce peak MeHg concentrations after flooding (Kelly et al. 1997; Hall and Louis 2004; Mailman et al. 2006; Willacker et al. 2016). With respect to long-term management of reservoirs, strategies that minimize large fluctuations in water levels between years and avoid drawdown of water storage during spring could minimize fish Hg. However, we recognize that these practices may be difficult with increasingly unpredictable precipitation patterns.

Though not unique to just reservoirs, management strategies in waterbodies aimed at altering redox conditions through oxygen or nitrate addition can also be utilized to reduce MeHg production (Matthews et al. 2013; McCord et al. 2016). Other management actions aimed at decreasing MeHg production can include reducing the size and/or development of an anoxic hypolimnion using lake mixers or selective water withdrawals (Rask et al. 2010; Perron et al. 2014; Zouabi-Aloui et al. 2015). In addition, fish species can be stocked or removed in order to promote food web conditions that can decrease MeHg concentrations in target fish populations (Lepak et al. 2012; Wolff et al. 2017).

## Urbanization

Urban areas cover a relatively small percentage of the Earth’s surface, but are home to more than half of the world’s population and often include some commercial and subsistence fishing in adjoining and downstream waterways (Murkin et al. 2003; Liu et al. 2014). Compared to rural/natural areas, concentrations of Hg have been shown to be elevated in urban air (Lindberg et al. 2007; Kim et al. 2009; Wip et al. 2013; Fu et al. 2015), soil/street dust (Manta et al. 2002; Ordonez et al. 2003; Eckley and Branfireun 2008a; Huang et al. 2012; Rodrigues et al. 2014), and waterbodies (Mason and Sullivan 1998; Rice 1999; Lawson et al. 2001; Clark and Benoit 2009; Barringer et al. 2010; Rowland et al. 2010; Tong et al. 2013; Deonarine et al. 2015; McKee and Gilbreath 2015). The level of Hg contamination in different urban areas can vary by over an order of magnitude, depending on the presence of current and historical industrial activities as well as local geographic and meteorological variables. In some residential and non-industrial urban areas, total Hg concentrations are not elevated compared to rural/natural areas (Naik and Hammerschmidt 2011; Fleck et al. 2016).

In addition to differences in total atmospheric Hg concentrations, the proportion of atmospheric Hg that is particulate-bound in urban areas can be greater than in non-urban settings, resulting in enhanced deposition (Fu et al. 2015). The enrichment of particulate-bound Hg in urban air is likely a function of the proximity to emission sources and/or reactions with other urban area pollutants, such as oxidizing agents and airborne particulate matter. The deposition and accumulation of atmospheric Hg on urban impervious surfaces (e.g., buildings, roads, parking lots) often occurs in association with a thin layer of organic film from the dry deposition of gas phase pollutants (Diamond et al. 2000; Gingrich and Diamond 2001). Hg deposited to urban surfaces is relatively labile and it is estimated that roughly half is released back to the air and the other half mobilized via runoff (Fig. 4). Due to the impervious nature of many urban surfaces, deposited Hg remains exposed to direct sunlight, resulting in photoreduction and evasion of Hg in urban areas (Gabriel et al. 2006; Eckley and Branfireun 2008a). Because of the high mobility of Hg in urban environments, the mass of Hg that remains on urban impervious surfaces can be very low even though the concentrations are high.

With the low infiltration capacity of urban landscapes, runoff often occurs as overland flow, which can effectively remove Hg associated with urban surfaces and entrain Hg-bearing street dust particles for transport to adjoining waterbodies (Vaze and Chiew 2003; Fulkerson et al. 2007; Eckley and Branfireun 2008b, 2009). In forested streams, dissolved NOM plays an important role in the transport of Hg in the dissolved phase (Brigham et al. 2009; Stoken et al. 2016); in contrast, the dissolved organic matter content can be low in urban areas, with particulate-bound Hg dominating overall mass transport (Hurley et al. 1998; Lawson et al. 2001; Lyons et al. 2006; Eckley and Branfireun 2008b).

While total Hg can be very elevated in urban waterbodies, MeHg concentrations in street dust, sediment, and water have been shown to be relatively low (Huang et al. 2012), as have MeHg concentrations in fish (Scudder 2009; Chalmers et al. 2014). In general, urban stormwater management focuses on the rapid conveyance of water away from the built environment, thus reducing water stagnation and the formation of anoxic conditions. With increased peak flows and erosion in urban waterways, conditions are significantly less favorable for methylation by anaerobic microbes in urban environments than in depositional environments. Higher nitrogen and lower dissolved NOM in urban streams may also contribute to the lower methylation potential in urban environments. Finally, the bioavailability of inorganic Hg for methylation in urban environments may be lower due to higher proportion of inorganic Hg bound to particles.

Low-impact development features and green infrastructure are common in new urban developments and are designed to reduce discharge to streams and improve water quality through particle settling. Examples include stormwater retention ponds, constructed wetlands, bioswales, permeable pavement, and green roofs. Retention ponds and other constructed wetlands have been shown to be very effective at reducing metal concentrations, including total Hg; however, they can also be net sources of MeHg (Stamenkovic et al. 2005; Rumbold and Fink 2006; Jang et al. 2010; Strickman and Mitchell 2017b). Similar to patterns observed in reservoirs, newly constructed retention ponds and wetlands differ considerably in their initial and long-term MeHg production capabilities as a function of age and organic matter content of the impounded soils (Sinclair et al. 2012; Strickman and Mitchell 2017b). The impact of MeHg from urban constructed wetlands depends on their scale and hydrological connectivity to other waterways; however, this has not yet been explored in the literature. In most circumstances, constructed urban wetlands are not likely to be a significant contributing source of MeHg to receiving water bodies.

## Rice production

Agricultural production of rice and other managed agricultural wetlands can be a source of MeHg that bioaccumulates in local wildlife and release MeHg to downstream waterways (Ackerman and Eagles-Smith 2010; Ackerman et al. 2010; Alpers et al. 2014; Rothenberg et al. 2014; Windham-Myers et al. 2014a, b). Moreover, rice consumption is another recently recognized MeHg exposure route for humans, especially for certain communities in Asia (Rothenberg et al. 2014). Rice is a major staple agricultural crop and provides the primary source of food energy for nearly half of the global population. Consequently, rice paddies are one of the most widely distributed land uses in certain regions of the world, such as South and East Asia (FAO 2002). Rice production methods can be grouped into broad categories that include irrigated rice, rainfed rice (rainfed lowland rice and rainfed upland rice), and flood-prone rice. Irrigated rice represents approximately 55 % of total area of rice cultivation and 77 % of the global rice production and often employs alternate wetting and drying cycles as a means to reduce freshwater consumption without decreasing yields (FAO 2013).

Rice cultivation can foster anaerobic and organic carbon-rich habitats that promote the growth of Hg-methylating microbes, resulting in conditions where MeHg can accumulate in rice crops (Meng et al. 2010, 2011). As a result, rice consumption has been shown to be the dominant pathway of MeHg exposure for certain communities (e.g., mining areas and certain inland areas of southern China) (Feng et al. 2007; Zhang et al. 2010; Li et al. 2012, 2015b). Elevated concentrations of MeHg in rice grains (up to 140 µg/kg) have been reported in Indonesia (Krisnayanti et al. 2012) and in different parts of China (e.g., (Horvat et al. 2003; Qiu et al. 2008; Meng et al. 2010, 2011, 2014a, b; Liang et al. 2015; Tang et al. 2015). MeHg bioaccumulates in rice more readily than inorganic Hg, with bioaccumulation factors for MeHg that are 800–40 000 times higher than those for inorganic Hg (Meng et al. 2010, 2011, 2014a; Zhang et al. 2010). MeHg exposure via rice in other global regions is also a possibility, as described in a recent study that found moderate amounts of mercury in European rice products (Brombach et al. 2017).

Rice seeds have the highest ability to accumulate MeHg compared to the other tissues of the plant (e.g., root, stalk, and leaf), and rice paddy soils are the principal source of MeHg taken up by these plants (Meng et al. 2010, 2011; Strickman and Mitchell 2017a). MeHg in soil can be taken up by plant roots and then translocated to the aboveground parts (leaf and stalk). In the premature plant, the majority of MeHg is stored in the leaf and stalk; however, most of this MeHg is transferred to the seed during the ripening period. On a mass basis, the majority of MeHg is found in edible white rice. During grain processing, most of the inorganic Hg (~ 78 %) is eliminated, but the majority of the MeHg remains in the food product (~80 %) (Meng et al. 2014a). MeHg in whole rice seeds as well as the edible components exists almost exclusively as CH_3_Hg^+^ bonded to cysteine-like structures (Li et al. 2010; Meng et al. 2014a), in the form of individual CH_3_Hg-(L-cysteinate) complexes or as part of larger proteins containing cysteine moieties. This MeHg-cysteine association behaves like a mobile nutrient and is actively transported to the endosperm during seed ripening (Meng et al. 2014a). We also note that the CH_3_Hg-(L-cysteinate) complex is thought to be responsible for the transfer of MeHg across the blood–brain and placental barriers (Kerper et al. 1992; Kajiwara et al. 1996; Simmons-Willis et al. 2002; Clarkson et al. 2007).

In addition to the concern for MeHg exposure via rice consumption, rice agriculture can also be a source of MeHg to downstream ecosystems. Net export of MeHg from rice fields has been estimated in some locations, although this phenomenon may vary within each growing season (Bachand et al. 2014; Windham-Myers et al. 2014b; Tanner et al. 2017).

Management strategies to reduce MeHg in rice must be balanced with the need to maximize crop production. Water management strategies such as intermittent flooding might alter anaerobic Hg-methylating microbes relative to continuously flooded rice fields (Rothenberg et al. 2011, 2014, 2016; Peng et al. 2012; Alpers et al. 2014; Wang et al. 2014). Selenium (Se)-enriched paddy soils have been shown to reduce MeHg production in paddy soils, which may be related to the formation of Hg-Se complexes in the rhizosphere (Zhang et al. 2012a, 2014a; Wang et al. 2016). Thus, Se amendments have been proposed as a means to reduce the absorption and accumulation of MeHg in rice grains in areas of high Hg contamination (Zhao et al. 2014; Wang et al. 2016). However, this approach requires caution due to the known impacts of Se on wildlife (Simmons and Wallschläger 2005). Different rice cultivars also vary considerably in grain concentrations of MeHg, suggesting that appropriate cultivar selection could reduce MeHg accumulation and exposure in Hg-contaminated areas (Peng et al. 2012; Rothenberg and Feng 2012; Li et al. 2013). The selection of non-rice food agricultural crops (e.g., corn, rape, tobacco, and cabbage) could be another solution, as these crops do not accumulate MeHg to the same extent as observed for rice (although inorganic Hg accumulation would still occur) (Qiu et al. 2008).

Future research on the impacts of rice production for MeHg release to the environment and rice consumption for human Hg exposure should examine geographically diverse areas, including Asian countries beyond China as well as other major continents where rice production and consumption is substantial. Such data are critical in assessing the potential health risks associated with rice cultivation in Hg-contaminated soils. While the source, distribution, and accumulation of MeHg in rice plants, as well as transport and transformation of Hg species within paddy fields, have been previously studied, the processes of Hg methylation in rice fields and the controlling factors are not fully understood. Furthermore, uptake and translocation pathways and the detoxification of MeHg in rice plant are still unknown. As is the case in other ecosystems, newly deposited Hg may be more readily transformed to MeHg in rice paddies than older Hg (Meng et al. 2010, 2011). However, the mechanisms behind this phenomenon are not well understood, and the link between MeHg concentrations in rice and atmospheric Hg deposition requires further investigation.

## Gold mining and other mining activities

Hg releases associated with mining can encompass a broad range of activities, including contemporary artisanal small-scale gold mining (ASGM), historical mines, and contemporary industrialized mines. ASGM generally involves the utilization of Hg (often imported from other regions) for amalgamation and extraction of gold and other precious metals from ore. ASGM relies on unmechanized gold recovery and in some cases small and heavy equipment use by individuals and small groups (rather than multinational corporate entities that operate industrialized mines). Historically, the same Hg amalgamation process was used at larger scale gold and silver mines (e.g., Sierra range of North America, Potosí). The legacy of these activities and the former Hg mine sites such as Almaden, Idrija, Huancavelica, and the coastal range of California have ongoing environmental inputs of Hg to downstream locations (Rytuba 2000; Foucher et al. 2009; Hagan et al. 2015; Jimenez-Moreno et al. 2016).

ASGM is currently active in more than 70 countries, where it is typically little acknowledged, unregulated, or illegal (Veiga et al. 2006; Telmer and Veiga 2009; Swenson et al. 2011; Reichelt-Brushett et al. 2017). Collectively this practice represents a major global emission source of Hg to the atmosphere, with estimates ranging from 20 to 37 % of total anthropogenic Hg emissions (Lacerda 2003; Pirrone et al. 2010; United Nations Environment Programme 2013). These estimations often rely on a mass balance approach for regional imports and exports and can have a high degree of uncertainty given the lack of reliable information (Malm 1998; Lacerda 2003; Li et al. 2009, 2014; Telmer and Veiga 2009; Grimaldi et al. 2015) and few examples of directly quantified Hg emissions (Amouroux et al. 1999; Balzino et al. 2015). ASGM also increases health risks for workers (Gibb and O’Leary 2014; Kristensen et al. 2014; Castilhos et al. 2015) and riparian populations downstream of mining sites (Lebel et al. 1997; Grandjean et al. 1999; Maurice-Bourgoin et al. 2000; Bastos et al. 2006; Diringer et al. 2015), as described in a companion paper (Eagles-Smith et al. 2018).

The type of ASGM techniques (such as material collection, processing, and disposal techniques) and the extent of accidental spills govern the amount of Hg release to the surrounding environment (Telmer and Veiga 2009; Balzino et al. 2015). Increases in local soil Hg content (Malm et al. 1995; van Straaten 2000) and soil erosion rates are common features of many ASGM areas (Swenson et al. 2011), and together are the key aspects influencing Hg transport and exposure to downstream communities. Extensive deforestation is often associated with ASGM and could further contribute to soil erosion and Hg export from watersheds. However, the impact of deforestation on Hg transport (relative to inadvertent release and deposition of Hg from the mining itself) requires further examination, perhaps by combining remote sensing data (e.g., satellite land cover) and field observations of Hg mobilization in watersheds (Swenson et al. 2011; Diringer et al. 2015; Lobo et al. 2016). The significance of terrestrial Hg inputs from ASGM relative to direct atmospheric deposition of Hg to waterbodies near ASGM (Fig. 3) could provide information for the assessment of other systems and insights for Hg exposure mitigation.

While ASGM and related processes (e.g., deforestation, soil erosion, urbanization) are known to increase the transport of Hg in watersheds, related impacts on biogeochemical transformations of Hg are also important to consider (Boudou et al. 2005; Alanoca et al. 2016). The chemical forms of Hg mobilized from recently deforested areas may differ from other terrestrial sources, and this difference may be relevant for Hg methylation and MeHg bioaccumulation. Some ASGM operations utilize cyanide in conjunction with Hg amalgamation, perhaps to reduce Hg usage or as a replacement for Hg. Environmental releases of cyanide have been hypothesized to alter methylation of Hg in downstream receiving waters perhaps by suppressing the biological activity of methylators (Tarras-Wahlberg et al. 2001; Guimaraes et al. 2011). This hypothesis remains to be fully investigated.

Compared to ASGM and historical mining, contemporary industrial mining operations are distinct in terms of their impacts on Hg cycling, most notably because they do not use Hg as part of the ore extraction process. However, these mines can increase mobilization of Hg if the orebody of interest is naturally enriched in Hg (typically gold mines as well as some copper and zinc mines). Hg can be released during ore processing via stack emissions and water discharges, but these are typically regulated and can be reduced through traditional pollution control technologies. However, fugitive surface-to-air fluxes from the ore, tailings, and waste rock piles can be a significant source of emissions from mines (Eckley et al. 2011a). Because of the large surface area covered by many contemporary industrial mines, the annual surface-to-air emissions scaled over an entire mine site can be > 100 kg/year (Eckley et al. 2011b). These surface emissions can be substantially reduced by capping mine waste with a thin layer of low-Hg topsoil and/or applying Hg control reagents to the mine wastes, though the latter approach needs further evaluation under field conditions (Eckley et al. 2011b; Miller and Gustin 2013).

In addition to direct emission of Hg to the air, mine operations can also impact Hg methylation in downstream aquatic systems by providing a substantial source of sulfate to receiving waterbodies (Berndt et al. 2016; Bailey et al. 2017) and by altering the surrounding hydrology as a result of dewatering around the mine pit/tunnels during operations and subsequent rewetting after closure (Willacker et al. 2016; Eckley et al. 2017). The drawdown and wetting cycles yield similar impacts on Hg methylation as that noted for reservoirs. After the stoppage of operations at open pit mines, the resulting deep pit lakes are susceptible to stratification, very high sulfate levels, and low organic carbon levels that have implications for MeHg production (Meier et al. 2012; Gammons et al. 2013).

Strategies to manage Hg release from historical and contemporary mining activities remain an ongoing challenge, mainly due to persistent and long-term inputs to ecosystems and broad geographic distribution of the impact. For example, some of the highest fish Hg concentrations in Canada are associated with areas where Hg was historically used to extract gold (Lockhart et al. 2005). Similarly, Hg pollution related to historical Hg mines and use of Hg for gold mining during the California gold rush during the mid-1800 s continues to be a concern for downstream waterbodies (May et al. 2000; Alpers et al. 2006). For contemporary ASGM, alternatives to Hg (such as cyanide and borax) have been suggested, but they also represent additional challenges, particularly for subsistence miners with limited resources (Hidayati et al. 2009; Spiegel and Veiga 2010; Velásquez-López et al. 2011; Veiga et al. 2014; Cordy et al. 2015; Køster-Rasmussen et al. 2016). The agreements outlined in the Minamata Convention may help to regulate Hg trade (see companion paper (Selin et al. 2018); however, the impact on unregulated Hg markets remains uncertain. Overall, effective management of contemporary ASGM requires a comprehensive approach that takes into consideration the environmental impacts of concurrent activities as well as socioeconomic constraints in applying management strategies.

## Industrial point sources and remediation

Historical Hg contamination from industrial sources represents a major challenge to address for site managers and neighboring communities who are vulnerable to Hg exposure. In addition to sites impacted by mining, other types of industrial Hg contamination include waste discharged from chlor-alkali processing facilities, pulp/paper mills, oil/gas production sites, and chemical production facilities. The age of the contamination at any single site is often several decades old (although newer industrial contamination still occurs in areas without close monitoring or established environmental regulations). Impacted sites include terrestrial sites as well as surface waters where Hg is typically concentrated in sediments (Lindeström 2001; Bloom et al. 2004; New York State Department of Environmental Conservation and U.S. Environmental Protection Agency 2005; Tomiyasu et al. 2006; Skyllberg et al. 2007; Ullrich et al. 2007a, b; Bravo et al. 2009, 2014; Balogh et al. 2015). Industrially impacted sites also include subsurface zones where groundwater discharge of Hg into surface waters is a concern (Flanders et al. 2010; Southworth et al. 2010). In many of these systems, total Hg content in soil and sediment is enriched by orders of magnitude relative to other ecosystems where Hg is also a concern (Fig. 1). In some exceptional cases, exposure to inorganic Hg from soil or vapors may pose a direct health risk (Robins et al. 2012; Hagan et al. 2013, 2015). Nevertheless, most industrially polluted sites raise concerns because of MeHg bioaccumulation, mobilization of Hg to downstream locations, and/or evasion of Hg to the atmosphere. We note that our search of the published literature on industrially contaminated sites revealed sediment MeHg contents that tended to be greater than other types of sites with comparable total Hg content (e.g., mining sites) (Fig. 1). This observation might be reflective of research activities that prioritize towards sites with potential health risks (i.e., sites with high levels of MeHg production and bioaccumulation) relative to other industrial sites with little MeHg impact.

The management of industrially contaminated locations requires assessment of Hg contamination and risk, followed by the development of strategies for remediation or long-term management (Randall and Chattopadhyay 2013; Bigham et al. 2017). In the course of developing a management and monitoring strategy, managers first quantify all sources of Hg to the system, identify areas of high exposure risk (e.g., high Hg or MeHg concentrations), and formulate benchmark goals for addressing the problems on the path towards a remedial action plan. These decisions require models (conceptual and/or numeric) that can be used to understand relevant processes for Hg fate, and perhaps, more importantly, delineate the uncertainties for risk. In many instances, the evaluation of a site may reveal that the risk of Hg is declining over time (e.g., due to the aging of Hg), and an understanding of these trends can help guide decisions to implement active or passive (and typically less disruptive) strategies for ecosystem recovery. Industrial sites can also have multiple pollutants, in addition to Hg, that drive exposure risks, and the best remediation and management decisions for one target pollutant could increase the risks posed by other targets.

Natural recovery of a system (with appropriate monitoring) is often the best option for large-scale industrial sites experiencing decreasing trends in Hg reactivity and bioavailability. This passive approach is often justified not only by its lower cost, but also by minimizing disruption to the ecosystem, which can be a negative consequence of more active remediation approaches. Active strategies include ex situ methods such as dredging and excavation for remediation of high priority Hg hotspots (Rudd et al. 2016). For sites of lower Hg enrichment and broad spatial extent, in situ methods have garnered substantial interest in recent years due to the inherently less disruptive impact of these methods on the surrounding ecosystem, the potential for long-term mitigation, and the reduction in costs relative to dredging and excavation (Ghosh et al. 2011; Wang et al. 2012). For example, sediment caps may be used for stream bank stabilization and erosion control (generally aiming to halt or minimize mobilization of Hg-bearing particles) (Wang et al. 2004; Johnson et al. 2010). In situ amendments involve addition of chemicals or materials directly to soil or sediments to alter the biogeochemical conditions of the site. For example, nitrate amendments and active oxygenation of reservoirs have successfully been used to artificially elevate the redox potential of the water column as a means to eliminate or bury the anaerobic zone that fosters MeHg producing microorganisms (Matthews et al. 2013; Beutel et al. 2014). Other amendments such as black carbon sorbents and ferrous iron aim to sequester Hg in sediment/soil and reduce the Hg and MeHg solubility and bioavailability for uptake (Mehrotra and Sedlak 2005; Ulrich and Sedlak 2010; Gilmour et al. 2013b; Gomez-Eyles et al. 2013). We note, however, that the impact of these processes on Hg speciation remains poorly understood. For example, the addition of activated carbon has been shown to decrease, increase, or not change overall levels of MeHg in sediment microcosms (Gilmour et al. 2013b). These responses might reflect reductions in Hg bioavailability for methylation, reduction of MeHg availability for demethylation, or alterations to the microbial community that are essential to these processes. Nevertheless, activated carbon amendments are a promising option in some cases for reducing MeHg bioavailability to benthic biota.

Many challenges remain in the development of effective Hg management strategies for industrially polluted sites. Innovative tools for source attribution and risk assessment are in great need and remain an active area of research. For cases where monitored natural attenuation of Hg is insufficient, in situ remediation technologies such as chemical amendments and sediment caps are promising; however, the implementation of these methods is, for the most part, currently limited to lab scale and pilot-scale testing. Full-scale implementation will require a better understanding of the long-term effects of in situ technologies as well as methods to evaluate site characteristics that inform the remediation selection process.

## Summary of management opportunities and research needs

A wide variety of anthropogenic and natural perturbations to landscapes have significant impacts on Hg cycling in watersheds (summarized in Table 1). These impacts are of concern if they increase Hg exposure risks to humans and wildlife. As such, water quality criteria for Hg tend to consist of fish-based MeHg content, rather than an aquatic or soil Hg/MeHg value. While this approach for regulation provides a direct connection between exposure and risk, this type of criterion does not inform clear ecosystem management strategies.

**Table 1:** Summary of landscape perturbations, how they influence methylmercury in biota, and potential interventions for site managers

Perturbations such as…	Change landscapes by	Impact Hg in the environment by	Have potential management strategies and interventions such as
Altered loading to surface waters	Variable input of Hg from direct atmospheric deposition and release from upland sources	Introducing multiple sources of Hg with a range of methylation potentials	Valuation of Hg inputs based on mass load, Hg speciation, and methylation potential Multiple avenues of control (e.g., nutrient loads, water column oxygenation) to reduce MeHg production and bioaccumulation
	Increasing sulfate inputs to freshwaters	Stimulating Hg-methylating bacteria, increasing sulfide that strongly binds Hg^2+^	
	Increasing nitrogen, phosphorous, and organic matter loads	Eutrophication, biodilution, and alteration in food web structure that increases MeHg in biota	
Forestry	Disturbance of soil cover by machinery	Increasing erosion of Hg-bearing soil particles	Logging practices to reduce erosion and soil disturbance Selection of logging sites less vulnerable to impacts Promotion of faster forest regeneration
	Increasing soil moisture	Increasing discharge, fluxes of Hg to downstream bodies of water, methylation in upland soils	
	Increasing organic carbon inputs from logging debris	Enhancing microbial activity and MeHg production	
Urbanization	Increasing impervious land surface cover	Reducing catchment retention of Hg and increasing mobilization in runoff	Stormwater management best practices
	Construction of retention ponds and wetlands	Increasing habitats that harbor Hg methylation processes	
Reservoirs	Flooding carbon-rich soils in newly formed reservoirs	Increase MeHg production and bioaccumulation within years after flooding	Site selection and preparation Water-level control Water column oxygenation and destratification
	For older reservoirs, fluctuations in water level and water quality	Create conditions that can increase MeHg in biota	
Rice cultivation	Creating conditions that favor Hg methylation in paddy soils	Enhanced bioaccumulation of MeHg in rice grains and exposure to certain populations	Water management Cultivar selection Chemical amendments to soil
Mining	Importing Hg for artisanal gold mining Deforestation	Increasing levels of Hg in soils and water and increase emissions to air	Managed mining concessions Controls on surface runoff and atmospheric emissions
		Increasing Hg in runoff and air emissions	
	Increasing sulfate loads in downstream areas	Increasing Hg methylation in freshwater ecosystems	
Industrial Hg use and releases	Release of Hg to surroundings	Increasing levels of Hg in soil and water; emissions to air	Monitored natural recovery to allow Hg to age in place Dredging and excavation of soil/sediment In situ caps and chemical amendments
	Long-term contamination of Hg from multiple sources	Creating variations in Hg methylation potential and bioavailability depending on source, age, and chemical form	

*Hg* mercury, *MeHg* monomethylmercury

As noted in the preceding sections, a specific remedial action or ecosystem perturbation requires the consideration of widely variable response times (e.g., years to decades) for the biogeochemical processes leading to MeHg bioaccumulation; understanding the time scales may be the critical factor in choosing any local Hg mitigation strategy. Knowledge of the relative sources of Hg input (e.g., surface versus terrestrial loading as shown in Fig. 3) could offer managers insights on the variety of Hg sources to a specific site, the relative response times for control of these sources, and ecosystem management strategies to minimize MeHg bioaccumulation.

Whole water or soil/sediment total Hg or MeHg concentration criteria might be helpful as a shorter-term gauge of management effectiveness, but sediment/soil Hg criteria could be misleading in that total Hg and MeHg concentrations do not necessarily correlate with MeHg bioaccumulation. As such, criteria based on a “bioavailable” fraction of total Hg, bioavailable MeHg, and net Hg methylation potential should be considered as we improve the functionality of metrics for water quality management.

Approaches for assessing the potential of Hg mobilization and net methylation at sites remain a challenge. However, recent gains in scientific knowledge could be utilized by site managers. For example, the mobilization of Hg to downstream and downgradient locations is often linked to the mobilization of particles (Flanders et al. 2010). Thus, Hg levels can be predicted by particle loadings. [We note that sites impacted by liquid elemental Hg^0^ contamination are an exception, where mobilization is influenced by dissolution and corrosion rates of discrete Hg^0^ phases in addition to transport of secondary mineral particles (Southworth et al. 2010).] Natural organic matter can facilitate particle transport by coating particles and reducing colloidal aggregation and deposition rates. Moreover, recent research has demonstrated that this effect varies with the quality of the NOM (e.g., molecular weight, chemical structure) (Deonarine et al. 2011; Philippe and Schaumann 2014; Louie et al. 2015, 2016). Optical properties of dissolved NOM such as specific UV absorbance and fluorescence signatures could enable the use of real-time sensors deployed in surface waters as proxies for dissolved and colloidal Hg transport (Dittman et al. 2009, 2010; Burns et al. 2013).

New tools are also in development to enable meaningful monitoring information of Hg transformation potential and source attribution. For example, stable Hg isotope signatures are a promising tool to delineate Hg inputs from multiple sources (Liu et al. 2011; Bartov et al. 2013; Deonarine et al. 2013; Sherman et al. 2015; Wiederhold et al. 2015). However, the application of Hg isotopes requires measurements of the appropriate “endmember” samples as well as an understanding of the extent of isotopic fractionation that could occur from a variety of biogeochemical transformation processes at the site. The deconvolution of fractionation processes represents the major hurdle in applying Hg isotopes information to source apportionment.

Recent advances in our understanding of microbial Hg methylation may enable the development of new tools to monitor and quantify net MeHg production potential at field sites and perhaps predict the impact of various remediation strategies. For example, the discovery of the *hgcAB* gene cluster in methylating microorganisms has led to new biomolecular tools to quantify the abundance, and perhaps activity of methylating microbes in nature (Gilmour et al. 2013a; Parks et al. 2013; Podar et al. 2015; Christensen et al. 2016).

Methods to quantify the reactivity of Hg and bioavailability of Hg to methylating microbes, such as chemical equilibrium models or selective extractions, have been tested over the years, but with limited success (Hsu-Kim et al. 2013; Ticknor et al. 2015). These results might be explained by the complexity of Hg-sulfide-NOM species in soil and sediment, which include nanostructured particulate phases of varying reactivity (e.g., dissolution potential) in anaerobic settings (as shown in Fig. 2). Hg uptake into methylating microbes is likely to involve an active membrane transport process (Schaefer et al. 2011, 2014), and not simply uptake of neutral Hg-sulfide solutes (Benoit et al. 1999). For this reason, as well as challenges in differentiating between dissolved solutes and colloidal particles in water samples, chemical equilibrium models of neutral Hg species are no longer employed by the scientific community to ascertain Hg bioavailability to methylating bacteria (Hsu-Kim et al. 2013).

Instead, alternative markers to quantify Hg reactivity and bioavailability are needed, especially models or biologically relevant measurements that can accommodate the spectrum of Hg species in soil and sediment and are consistent with the process by which methylating microbes take up Hg (Ticknor et al. 2015). While several methods to quantify a nominally “reactive” fraction of Hg have been proposed (Bloom et al. 2003; Marvin-DiPasquale et al. 2009; Liang et al. 2013; Ticknor et al. 2015), the measurements must be interpreted with great care because this reactivity may not necessarily be relevant to processes influencing bioavailability. Moreover, these methods generally have not been explicitly evaluated in experiments that compare reactivity and Hg bioavailability for methylators. Likewise, the use of stable isotope spike experiments to assess the net Hg methylation rate potential has uncertainties because the outcome of these assays are highly subjective towards methodology (i.e., chemical form of the Hg spike, incubation time and conditions) and unknown changes in speciation of the Hg spike in relation to the form of ambient Hg at the site.

While tremendous progress has been made in understanding the process of microbial Hg methylation, the mechanism of Hg uptake, how the rates of Hg methylation vary among the diverse species of methylating microbes, and if their abundance or activity correlate to methylation rates remain unknown. The process of dark MeHg degradation in benthic zones remains an even greater mystery and requires more attention by the research community, especially since remediation of contaminated sites might need to focus on strategies to enhance MeHg degradation. With progress in the understanding of processes that contribute to Hg mobilization and net MeHg production, novel methods for effective and lasting remediation and monitoring technologies are within reach.

Transformational improvements to Hg site management, such as those outlined above, will take time, but the need for comprehensive risk assessment tools remain urgent. Typical site assessments attempt to account for all Hg inputs and outputs in the system, which enables an understanding of primary sources and sinks. Other parameters such as the concentrations and fluxes of sulfate, organic carbon, filter-passing Hg and MeHg fractions, dissolved and total sulfide, and redox potential, among others, could provide insight into the factors controlling rates of MeHg production, degradation, and food web bioaccumulation and biomagnification. In some cases, measurements of isotope ratios of Hg, the bioavailable fraction of Hg, and the soil/water microbiomes might be justified as the development and applications of these tools improve with additional research. Advanced data analysis techniques should be utilized to help us discern large datasets of parameters interlinked by non-linear and poorly defined relationships.

Global-scale perturbations such as climate change will have local-scale effects such as rising sea level, melting tundra soils, and altered and extreme precipitation regimes. Impacts could include large releases of soluble organic matter, mobilization of Hg-bearing particles, and redox fluctuations and gradients, all of which can ultimately impact MeHg bioaccumulation as discussed in previous sections.

Overall, this synthesis paper outlined the effects of major anthropogenic landscape perturbations on the distribution and methylation of Hg in aquatic ecosystems. Much of the research over recent decades has had a relatively narrow geographical focus (e.g., North America, Europe, parts of Asia); however in other locations (Latin America, Africa, Asia Pacific), large populations continue to be vulnerable to the negative health consequences of MeHg exposure. Insights from the existing research literature can provide insights into the development of substantive approaches to mitigate Hg distribution and exposure in understudied regions. Regardless, research in these regions is still sorely needed, particularly in tropical environments that have received much less attention in the Hg biogeochemical research field. Altogether, global- and local-scale perturbations to landscapes alter the transport and transformations of Hg in complex ways, and an understanding of this complexity is needed to guide international and regional efforts to manage and monitor reductions in MeHg exposure to populations.

## Electronic supplementary material

Below is the link to the electronic supplementary material.

Supplementary material 1 (PDF 182 kb)
