# Supplementary material for: Challenges and opportunities for managing aquatic mercury pollution in altered landscapes
Source: Ambio. 2018 Jan 31;47(2):141–69. doi: 10.1007/s13280-017-1006-7 (PMC5794684; doi:10.1007/s13280-017-1006-7)
Supplement: Supplementary file 1 — Supplementary material 1 (PDF 182 kb) [file 13280_2017_1006_MOESM1_ESM.pdf]

Ambio

Electronic Supplementary Material
This supplementary material has not been peer-reviewed.

Title: **Challenges and Opportunities for Managing Aquatic Mercury Pollution in Altered Landscapes**

Authors: Heileen Hsu-Kim, Chris S. Eckley, Dario Achá, Xinbin Feng, Cynthia C. Gilmour, Sofi Jonsson, Carl P.J. Mitchell

Contents:

Supporting Information for Figure 1
Supporting Information for Figure 3
    Table S1: Terrestrial Hg inputs relative to direct atmospheric input data for Figure 3 and source citations
Explanatory information for Figure 4
References

Supporting Information for Figure 1

The data points presented in Figure 1 are based on a review of the literature reporting measurements of sediment total Hg and MeHg contents at locations impacted by different landscape perturbations (mining, industrial activity, rice production, and urbanization). Only a very limited number of sediment total Hg and MeHg data were available from streams impacted by forestry operations; thus, thse data were not included in the figure.

In papers where multiple data points were presented from a similar area (e.g., data from multiple years/seasons and/or to identify within-site spatial variability), we calculated a median value and included this value to represent that specific site in Figure 1. Some papers reported only mean values, and this value was plotted in Figure 1.

Urban Areas

The urban data (n=121) presented in Figure 1 are from following papers:

Chalmers, A.T., Krabbenhoft, D.P., Van Metre, P.C., Nilles, M.A., 2014. Effects of urbanization on mercury deposition and New England. Environmental Pollution 192, 104-112.

Deonarine, A., Hsu-Kim, H., Zhang, T., Cai, Y., Richardson, C.J., 2015. Legacy source of mercury in an urban stream-wetland ecosystem in central North Carolina, USA. Chemosphere 138, 960-965.

* Fleck, J.A., Marvin-DiPasquale, M., Eagles-Smith, C.A., Ackerman, J.T., Lutz, M.A., Tate, M., Alpers, C.N., Hall, B.D., Krabbenhoft, D.P., Eckley, C.S., 2016. Mercury and methylmercury in aquatic sediment across western North America. Science of the Total Environment 568, 727-738.
* Includes data sourced from multiple other studies

Heyes, A., Miller, C., Mason, R.P., 2004. Mercury and methylmercury in Hudson River sediment: impact of tidal resuspension on partitioning and methylation. Marine Chemistry 90, 75-89.

Kim, E., Noh, S., Lee, Y.G., Kundu, S.R., Lee, B.G., Park, K., Han, S., 2014. Mercury and methylmercury flux estimation and sediment distribution in an industrialized urban bay. Marine Chemistry 158, 59-68.

Marvin-DiPasquale, M., Lutz, M.A., Brigham, M.E., Krabbenhoft, D.P., Aiken, G.R., Orem, W.H., Hall, B.D., 2009. Mercury Cycling in Stream Ecosystems. 2. Benthic Methylmercury Production and Bed Sediment-Pore Water Partitioning. Environmental Science & Technology 43, 2726-2732.

Scudder, B.C., Chasar, L.C., Wentz, D.A., Bauch, N.J., Brigham, M.E., Moran, P.W., and Krabbenhoft, D.P, 2009. Mercury in fish, bed sediment, and water from streams across the United States, 1998–2005, U.S. Geological Survey Scientific Investigations Report USGS, p. 74.

Sinclair, K.A., Xie, Q., Mitchell, C.P.J., 2012. Methylmercury in water, sediment, and invertebrates in created wetlands of Rouge Park, Toronto, Canada. Environmental Pollution 171, 207-215.

Strickman, R.J., Mitchell, C.P.J., 2017. Methylmercury production and accumulation in urban stormwater ponds and habitat wetlands. Environmental Pollution 221, 326-334.

Yang, J., Chen, L., Shi, W.L., Liu, L.Z., Li, Y., Meng, X.Z., 2015. Mercury distribution in sediment along urban-rural gradient around Shanghai (China): implication for pollution history. Environmental Science and Pollution Research 22, 1697-1704.

Industrial Areas

The industrial area data (n=33) in Figure 1 are from the following papers:

Balcom, P.H., Schartup, A.T., Mason, R.P., Chen, C.Y., 2015. Sources of water column methylmercury across multiple estuaries in the Northeast US. Marine Chemistry 177, 721-730.

Balogh, S.J., Tsui, M.T.K., Blum, J.D., Matsuyama, A., Woerndle, G.E., Yano, S., Tada, A., 2015. Tracking the Fate of Mercury in the Fish and Bottom Sediments of Minamata Bay, Japan, Using Stable Mercury Isotopes. Environmental Science & Technology 49, 5399-5406.

Birkett, J.W., Lester, J.N., 2005. Distribution of mercury and methylmercury in the sediments of a lowland river system. Proceedings of the Royal Society a-Mathematical Physical and Engineering Sciences 461, 1335-1355.

Bloom, N.S., Moretto, L.M., Ugo, P., 2004. A comparison of the speciation and fate of mercury in two contaminated coastal marine ecosystems: The Venice Lagoon (Italy) and Lavaca Bay (Texas). Limnology and Oceanography 49, 367-375.

Bravo, A.G., Cosio, C., Amouroux, D., Zopfi, J., Cheualley, P.A., Spangenberg, J.E., Ungureanu, V.G., Dominik, J., 2014. Extremely elevated methyl mercury levels in water, sediment and organisms in a Romanian reservoir affected by release of mercury from a chlor-alkali plant. Water Research 49, 391-405.

Bravo, A.G., Loizeau, J.L., Ancey, L., Ungureanu, V.G., Dominik, J., 2009. Historical record of mercury contamination in sediments from the Babeni Reservoir in the Olt River, Romania. Environmental Science and Pollution Research 16, 66-75.

Buckman, K.L., Marvin-DiPasquale, M., Taylor, V.F., Chalmers, A., Broadley, H.J., Agee, J., Jackson, B.P., Chen, C.Y., 2015. Influence of a chlor-alkali superfund site on mercury bioaccumulation in periphyton and low-trophic level fauna. Environmental Toxicology and Chemistry 34, 1649-1658.

Campbell, K.R., Ford, C.J., Levine, D.A., 1998. Mercury distribution in Poplar Creek, Oak Ridge, Tennessee, USA. Environmental Toxicology and Chemistry 17, 1191-1198.

Flanders, J.R., Turner, R.R., Morrison, T., Jensen, R., Pizzuto, J., Skalak, K., Stahl, R., 2010. Distribution, behavior, and transport of inorganic and methylmercury in a high gradient stream. Applied Geochemistry 25, 1756-1769.

Huffman, R.L., Wagner, R.J., Toft, J., Cordell, J., DeWild, J.F., Dinicola, R.S., Aiken, G.R., Krabbenhoft, D.P., Marvin-DiPasquale, M., Stewart, A.R., Moran, P.W., and Paulson, A.J., , 2012. Mercury species and other selected constituent concentrations in water, sediment, and biota of Sinclair Inlet, Kitsap County, Washington, 2007–10: U.S. Geological Survey Data Series 658, 64 p.

Janssen, S.E., Johnson, M.W., Blum, J.D., Barkay, T., Reinfelder, J.R., 2015. Separation of monomethylmercury from estuarine sediments for mercury isotope analysis. Chemical Geology 411, 19-25.

Kim, E., Noh, S., Lee, Y.G., Kundu, S.R., Lee, B.G., Park, K., Han, S., 2014. Mercury and methylmercury flux estimation and sediment distribution in an industrialized urban bay. Marine Chemistry 158, 59-68.

Liu, B., Yan, H.Y., Wang, C.P., Li, Q.H., Guedron, S., Spangenberg, J.E., Feng, X.B., Dominik, J., 2012. Insights into low fish mercury bioaccumulation in a mercury-contaminated reservoir, Guizhou, China. Environmental Pollution 160, 109-117.

The Penobscot River Mercury Study Panel. 2013. Mercury Contamination of the Penobscot River Estuary: Current Situation, Remediation Targets and Possible Remediation Procedures. NYSDEC, US EPA Region-2. 2005. Onondaga Lake Bottom Subsite of the Onondaga Lake Superfund Site.

Scudder, B.C., Chasar, L.C., Wentz, D.A., Bauch, N.J., Brigham, M.E., Moran, P.W., and Krabbenhoft, D.P, 2009. Mercury in fish, bed sediment, and water from streams across the United States, 1998–2005, U.S. Geological Survey Scientific Investigations Report USGS, p. 74.

Skyllberg, U., Drott, A., Lambertsson, L., Bjorn, E., Karlsson, T., Johnson, T., Heinemo, S.A., Holmström, H., 2007. Net methylmercury production as a basis for improved risk assessment of mercury-contaminated sediments. Ambio 36, 437-442.

Tomiyasu, T., Matsuyama, A., Eguchi, T., Fuchigami, Y., Oki, K., Horvat, M., Rajar, R., Akagi, H., 2006.Spatial variations of mercury in sediment of Minamata Bay, Japan. Science of the Total Environment 368, 283-290.

Rice/Agricultural Areas

The rice/agricultural data (n=6) presented in Figure 1 are from following papers:

Marvin-DiPasquale, M., Windham-Myers, L., Agee, J.L., Kakouros, E., Kieu, L.H., Fleck, J.A., Alpers, C.N., Stricker, C.A., 2014. Methylmercury production in sediment from agricultural and non-agricultural wetlands in the Yolo Bypass, California, USA. Science of the Total Environment 484, 288-299.

Meng, B., Feng, X.B., Qiu, G.L., Cai, Y., Wang, D.Y., Li, P., Shang, L.H., Sommar, J., 2010. Distribution Patterns of Inorganic Mercury and Methylmercury in Tissues of Rice (Oryza sativa L.) Plants and Possible Bioaccumulation Pathways. Journal of Agricultural and Food Chemistry 58, 4951-4958.

Meng, B., Feng, X.B., Qiu, G.L., Liang, P., Li, P., Chen, C.X., Shang, L.H., 2011. The Process of Methylmercury Accumulation in Rice (Oryza sativa L.). Environmental Science & Technology 45, 2711-2717.

Windham-Myers, L., Fleck, J.A., Ackerman, J.T., Marvin-DiPasquale, M., Stricker, C.A., Heim, W.A., Bachand, P.A.M., Eagles-Smith, C.A., Gill, G., Stephenson, M., Alpers, C.N., 2014. Mercury cycling in agricultural and managed wetlands: A synthesis of methylmercury production, hydrologic export, and bioaccumulation from an integrated field study. Science of the Total Environment 484, 221-231.

Xu, X.H., Meng, B., Zhang, C., Feng, X.B., Gu, C.H., Guo, J.Y., Bishop, K., Xu, Z.D., Zhang, S.S., Qiu, G.L., 2017. The local impact of a coal-fired power plant on inorganic mercury and methyl-mercury distribution in rice (Oryza sativa L.). Environmental Pollution 223, 11-18.

Zhang, H., Feng, X.B., Larssen, T., Shang, L., Li, P., 2010. Bioaccumulation of Methylmercury versus Inorganic Mercury in Rice (Oryza sativa L.) Grain. Environmental Science & Technology 44, 4499-4504.

Mining Impacted Areas

The mining-impacted area data (n=89) presented in Figure 1 are from following papers:

Diringer, S.E., Feingold, B.J., Ortiz, E.J., Gallis, J.A., Araujo-Flores, J.M., Berky, A., Pan, W.K.Y., Hsu-Kim, H., 2015. River transport of mercury from artisanal and small-scale gold mining and risks for dietary mercury exposure in Madre de Dios, Peru. Environmental Science-Processes & Impacts 17, 478- 487.

Feng, X.B., Dai, Q.Q., Qiu, G.L., Li, G.H., He, L., Wang, D.Y., 2006. Gold mining related mercury contamination in Tongguan, Shaanxi Province, PR China. Applied Geochemistry 21, 1955-1968.

* Fleck, J.A., Marvin-DiPasquale, M., Eagles-Smith, C.A., Ackerman, J.T., Lutz, M.A., Tate, M., Alpers, C.N., Hall, B.D., Krabbenhoft, D.P., Eckley, C.S., 2016. Mercury and methylmercury in aquatic sediment across western North America. Science of the Total Environment 568, 727-738.
 * Includes data sourced from multiple other studies

Li, B., Shi, J.B., Wang, X., Meng, M., Huang, L., Qi, X.L., He, B., Ye, Z.H., 2013. Variations and constancy of mercury and methylmercury accumulation in rice grown at contaminated paddy field sites in three Provinces of China. Environmental Pollution 181, 91-97.

Meng, B., Feng, X.B., Qiu, G.L., Cai, Y., Wang, D.Y., Li, P., Shang, L.H., Sommar, J., 2010. Distribution Patterns of Inorganic Mercury and Methylmercury in Tissues of Rice (Oryza sativa L.) Plants and Possible Bioaccumulation Pathways. Journal of Agricultural and Food Chemistry 58, 4951-4958.

Meng, B., Feng, X.B., Qiu, G.L., Liang, P., Li, P., Chen, C.X., Shang, L.H., 2011. The Process of Methylmercury Accumulation in Rice (Oryza sativa L.). Environmental Science & Technology 45, 2711-2717.

Meng, M., Li, B., Shao, J.-j., Wang, T., He, B., Shi, J.-b., Ye, Z.-h., Jiang, G.-b., 2014. Accumulation of total mercury and methylmercury in rice plants collected from different mining areas in China. Environmental Pollution 184, 179-186.

Molina, C.I., Gibon, F.M., Duprey, J.L., Dominguez, E., Guimaraes, J.R.D., Roulet, M., 2010. Transfer of mercury and methylmercury along macroinvertebrate food chains in a floodplain lake of the Beni River, Bolivian Amazonia. Science of the Total Environment 408, 3382-3391.

Qiu, G.L., Feng, X.B., Jiang, G.B., 2012. Synthesis of current data for Hg in areas of geologic resource extraction contamination and aquatic systems in China. Science of the Total Environment 421, 59-72.

Qiu, G.L., Feng, X.B., Wang, S.F., Shang, L.H., 2005. Mercury and methylmercury in riparian soil, sediments, mine-waste calcines, and moss from abandoned Hg mines in east Guizhou province, southwestern China. Applied Geochemistry 20, 627-638.

Rimondi, V., Bardelli, F., Benvenuti, M., Costagliola, P., Gray, J.E., Lattanzi, P., 2014. Mercury speciation in the Mt. Amiata mining district (Italy): Interplay between urban activities and mercury contamination. Chemical Geology 380, 110-118.

Rothenberg, S.E., Feng, X., Zhou, W., Tu, M., Jin, B., You, J., 2012. Environment and genotype controls on mercury accumulation in rice (Oryza sativa L.) cultivated along a contamination gradient in Guizhou, China. Science of the Total Environment 426, 272-280.

Scudder, B.C., Chasar, L.C., Wentz, D.A., Bauch, N.J., Brigham, M.E., Moran, P.W., and Krabbenhoft, D.P, 2009. Mercury in fish, bed sediment, and water from streams across the

United States, 1998–2005, U.S. Geological Survey Scientific Investigations Report USGS, p. 74.
Zhang, H., Feng, X.B., Larssen, T., Shang, L., Li, P., 2010. Bioaccumulation of Methylmercury versus Inorganic Mercury in Rice (Oryza sativa L.) Grain. Environmental Science & Technology 44, 4499-4504.

Reservoirs

The reservoir data (n=23) presented in Figure 1 are from following papers:

Becker, J.C., Groeger, A.W., Nowlin, W.H., Chumchal, M.M., Hahn, D., 2011. Spatial variability in the speciation and bioaccumulation of mercury in an arid subtropical reservoir ecosystem. Environmental Toxicology and Chemistry 30, 2300-2311.
Berzas Nevado, J.J., Rodriguez Martin-Doimeadios, R.C., Jimenez Moreno, M., 2009. Mercury speciation in the Valdeazogues River-La Serena Reservoir system: Influence of Almadein (Spain) historic mining activities. Science of the Total Environment 407, 2372-2382.
Caldwell, C.A., Canavan, C.M., Bloom, N.S., 2000. Potential effects of forest fire and storm flow on total mercury and methylmercury in sediments of an arid-lands reservoir. Science of the Total Environment 260, 125-133.
Castelle, S., Schafer, J., Blanc, G., Audry, S., Etcheber, H., Lissalde, J.-P., 2007. 50-year record and solid state speciation of mercury in natural and contaminated reservoir sediment. Applied Geochemistry 22, 1359-1370.
Feng, X., Jiang, H., Qiu, G., Yan, H., Li, G., Li, Z., 2009. Geochemical processes of mercury in Wujiangdu and Dongfeng reservoirs, Guizhou, China. Environmental Pollution 157, 2970-2984.
Gilmour, C.C., Henry, E.A., Mitchell, R., 1992. Sulfate Stimulation of mercury methylation in fresh-water sediments. Environmental Science & Technology 26, 2281-2287.
Gray, J.E., Hines, M.E., 2009. Biogeochemical mercury methylation influenced by reservoir eutrophication, Salmon Falls Creek Reservoir, Idaho, USA. Chemical Geology 258, 157-167.
Gray, J.E., Hines, M.E., Goldstein, H.L., Reynolds, R.L., 2014. Mercury deposition and methylmercury formation in Narraguinnep Reservoir, southwestern Colorado, USA. Applied Geochemistry 50, 82-90.
He, T., Zhu, Y., Yin, D., Luo, G., An, Y., Yan, H., Qian, X., 2015. The impact of acid mine drainage on the methylmercury cycling at the sediment-water interface in Aha Reservoir, Guizhou, China. Environmental Science and Pollution Research 22, 5124-5138.
Liu, B., Yan, H.Y., Wang, C.P., Li, Q.H., Guedron, S., Spangenberg, J.E., Feng, X.B., Dominik, J., 2012. Insights into low fish mercury bioaccumulation in a mercury-contaminated reservoir, Guizhou, China. Environmental Pollution 160, 109-117.
Liu, J., Jiang, T., Huang, R., Wang, D., Zhang, J., Qian, S., Yin, D., Chen, H., 2017. A simulation study of inorganic sulfur cycling in the water level fluctuation zone of the Three Gorges Reservoir, China and the implications for mercury methylation. Chemosphere 166, 31-40.
Meng, B., Feng, X., Qiu, G., Li, Z., Yao, H., Shang, L., Yan, H., 2016. The impacts of organic matter distribution and methylation on mercury in a hydroelectric reservoir in Wuijiang River, Southwest China. Environmental Toxicology and Chemistry 35, 191-199.
Mucci, A., Lucotte, M., Montgomery, S., Plourde, Y., Pichet, P., VanTra, H., 1995. Mercury remobilization from flooded soils in a hydroelectric reservoir of northern Quebec, La

Grande-2: Results of a soil resuspension experiment. Canadian Journal of Fisheries and Aquatic Sciences 52, 2507-2517.
Muresan, B., Cossa, D., Richard, S., Dominique, Y., 2008. Monomethylmercury sources in a tropical artificial reservoir. Applied Geochemistry 23, 1101-1126.
Rolfhus, K.R., Wiener, J.G., Haro, R.J., Sandheinrich, M.B., Bailey, S.W., Seitz, B.R., 2015. Mercury in streams at Grand Portage National Monument (Minnesota, USA): Assessment of ecosystem sensitivity and ecological risk. Science of the Total Environment 514, 192-201.
St Louis, V.L., Rudd, J.W.M., Kelly, C.A., Beaty, K.G., Bloom, N.S., Flett, R.J., 1994. Importance of wetlands as sources of methylmercury to boreal forest ecosystems. Canadian Journal of Fisheries and Aquatic Sciences 51, 1065-1076.
Tremblay, A., Cloutier, L., Lucotte, M., 1998. Total mercury and methylmercury fluxes via emerging insects in recently flooded hydroelectric reservoirs and a natural lake. Science of the Total Environment 219, 209-221.
Tremblay, A., Lucotte, M., 1997. Accumulation of total mercury and methyl mercury in insect larvae of hydroelectric reservoirs. Canadian Journal of Fisheries and Aquatic Sciences 54, 832-841.
Wang, F., Zhang, J., 2013. Mercury contamination in aquatic ecosystems under a changing environment: Implications for the Three Gorges Reservoir. Chinese Science Bulletin 58, 141-149.
Yan, H.Y., Li, Q.H., Meng, B., Wang, C.P., Feng, X.B., He, T.R., Dominik, J., 2013. Spatial distribution and methylation of mercury in a eutrophic reservoir heavily contaminated by mercury in Southwest China. Applied Geochemistry 33, 182-190.

Supporting Information for Figure 3

Table S1. Ratio of estimated Hg mass inputs from terrestrial sources (e.g., river, runoff, groundwater intrusion) relative to direct atmospheric deposition to surface water for a variety of aquatic systems. Ecosystems with large terrestrial Hg:atmospheric Hg input ratios are expected to respond more slowly to anticipated reductions in global Hg emissions, while ecosystems with low terrestrial Hg:atmospheric Hg input ratios that are expected to respond more quickly. Modified from (Jonsson 2013).

System	Terrestrial Hg: Atmospheric Hg Input Ratio	Reference
**Ocean**		
Global ocean (preindustrial)	0.030	Mason and Sheu 2002
Global ocean (current)	0.065, 0.10	Mason and Sheu 2002, Amos et al. 2013
Baltic Sea	0.53	Soerensen et al. 2016
Mediterranean Sea	0.70	Rajar et al. 2007
Arctic Ocean	2.7	Soerensen et al. 2016
**Estuaries**		
Global estuaries	1	Rolfhus and Fitzgerald 1995
San Francisco Bay (USA)	1.5	MacLeod et al. 2005
Tokyo Bay (Japan)	1.9	Sakata et al. 2006
Long Island Sound (USA)	7.9	Balcom et al. 2004
NY/NJ Harbor Estuary (USA)	80	Balcom et al. 2008
Chesapeake Bay (USA)	1.6	Mason et al. 1999
**Lakes and Reservoirs**		
Lake Superior (N. America)	0.63	Qureshi et al. 2009
Lake Michigan (USA)	0.31	Qureshi et al. 2009
Little Rock Lake (USA)	0.06	Watras et al. 1996, Qureshi et al. 2009
Lake Champlain (N. America)	1.6	Gao et al. 2006
Big Dam West (Canada)	11	Ethier et al. 2008
Spring Lake (USA)	0.088	Hines and Brezonik 2007
Lake Melville (Canada)	13	Schartup et al. 2015
Dongfeng Reservoir (China)	68	Feng et al. 2009
Wujiangdu Reservoir (China)	27	Feng et al. 2009
Petit-Saut Reservoir (French Guiana)	17	Muresan et al. 2007
Onondaga Lake (USA; historical industrial point source)	310	Qureshi et al. 2009
Clear Lake (USA; historical mining point source)	67 – 586	Suchanek et al. 2009

Exaplanatory Information for Figure 4

The runoff from forested catchments was estimated based on the average runoff ratio from 11 catchments described in the following papers: Mason et al. (1997), Balogh et al. (2005), Shanley et al. (2008), Domagalski et al. (2016). Surface emissions from forested catchments were estimated for each of the catchments where runoff data were available and varied depending on the forest type (marine West Coast coniferous, northwest mountains coniferous, or Great Lakes region deciduous), as described in Denkenberger et al. (2012), Eckley et al. (2016). Runoff from logged/harvested catchments was estimated by determining the increase in runoff Hg loads from nine harvested catchments relative to proximate reference/forested catchments described in Porvari et al. (2003), Allan (2009), Sorensen et al. (2009), de Wit et al. (2014), Eklof et al. (2014), Kronberg et al. (2016). The average increase in response to forest harvesting was multiplied by the runoff yield from forested sites described above. Surface emissions from harvested catchments were based on averaged data described in Eckley et al. (2016), Mazur et al. (2014), Eagles-Smith et al. (2016).

Runoff from urban catchments was estimated based on the average runoff ratio from nine urban catchments described in the following papers: Lawson et al. (2001), Eckley and Branfireun (2008), Naik and Hammerschmidt (2011), Domagalski et al. (2016). Emissions from urban surfaces were based on data obtained from six urban sites that are summarized in Denkenberger et al. (2012).

Runoff from catchments containing mining operations were based on the average from eight catchments that contained abandoned mines as summarized in Domagalski et al. (2016). Surface emissions from mine sites were based on the average data from five active and abandoned mining areas described in Engle et al. (2001), Nacht et al. (2004), Eckley et al. (2011), Kocman and Horvat (2011).

All runoff and emission values were scaled to a common deposition rate of 10 µg m^{-2} value to allow comparisons of the effect of different land-use perturbations between studies. The net landscape sink or source values were based on the difference between Hg inputs from deposition and Hg outputs from runoff and surface emissions.

References

Allan, C. J., A. Heyes, and R. J. Mackereth. 2009. Changes to groundwater and surface water Hg transport following clearcut logging: a Canadian case study. Royal Swedish Academy of Agriculture and Forestry **148**:50–54.

Amos, H. M., D. J. Jacob, D. G. Streets, and E. M. Sunderland. 2013. Legacy impacts of all-time anthropogenic emissions on the global mercury cycle. Global Biogeochemical Cycles **27**:410-421.

Balcom, P. H., W. F. Fitzgerald, G. M. Vandal, C. H. Lamborg, K. R. Rolfhus, C. S. Langer, and C. R. Hammerschmidt. 2004. Mercury sources and cycling in the Connecticut River and Long Island Sound. Marine Chemistry **90**:53-74.

Balcom, P. H., C. R. Hammerschmidt, W. F. Fitzgerald, C. H. Lamborg, and J. S. O'Connor. 2008. Seasonal distributions and cycling of mercury and methylmercury in the waters of New York/New Jersey Harbor Estuary. Marine Chemistry **109**:1-17.

Balogh, S. J., Y. H. Nollet, and H. J. Offerman. 2005. A comparison of total mercury and methylmercury export from various Minnesota watersheds. Science of the Total Environment **340**:261-270.

de Wit, H. A., A. Granhus, M. Lindholm, M. J. Kainz, Y. Lin, H. F. V. Braaten, and J. Blaszczak. 2014. Forest harvest effects on mercury in streams and biota in Norwegian boreal catchments. Forest Ecology and Management **324**:52-63.

Denkenberger, J. S., C. T. Driscoll, B. A. Branfireun, C. S. Eckley, M. Cohen, and P. Selvendiran. 2012. A synthesis of rates and controls on elemental mercury evasion in the Great Lakes Basin. Environmental Pollution **161**:291-298.

Domagalski, J., M. S. Majewski, C. N. Alpers, C. S. Eckley, C. A. Eagles-Smith, L. Schenk, and S. Wherry. 2016. Comparison of mercury mass loading in streams to atmospheric deposition in watersheds of Western North America: Evidence for non-atmospheric mercury sources. Science of the Total Environment **568**:638-650.

Eagles-Smith, C. A., J. G. Wiener, C. S. Eckley, J. J. Willacker, D. C. Evers, M. Marvin-DiPasquale, D. Obrist, J. A. Fleck, G. R. Aiken, J. M. Lepak, A. K. Jackson, J. P. Webster, A. R. Stewart, J. A. Davis, C. N. Alpers, and J. T. Ackerman. 2016. Mercury in western North America: A synthesis of environmental contamination, fluxes, bioaccumulation, and risk to fish and wildlife. Science of the Total Environment **568**:1213-1226.

Eckley, C. S., and B. Branfireun. 2008. Mercury mobilization in urban stormwater runoff. Science of the Total Environment **403**:164-177.

Eckley, C. S., M. Gustin, M. B. Miller, and F. Marsik. 2011. Scaling Non-Point-Source Mercury Emissions from Two Active Industrial Gold Mines: Influential Variables and Annual Emission Estimates. Environmental Science & Technology **45**:392-399.

Eckley, C. S., M. T. Tate, C.-J. Lin, M. Gustin, S. Dent, C. Eagles-Smith, M. A. Lutz, K. P. Wickland, B. Wang, J. E. Gray, G. C. Edwards, D. P. Krabbenhoft, and D. B. Smith. 2016. Surface-airmercury fluxes across Western North America: A synthesis of spatial trends and controlling variables. Science of the Total Environment **568**:651-665.

Eklof, K., J. Schelker, R. Sorensen, M. Meili, H. Laudon, C. von Bromssen, and K. Bishop. 2014. Impact of Forestry on Total and Methyl-Mercury in Surface Waters: Distinguishing Effects of Logging and Site Preparation. Environmental Science & Technology **48**:4690-4698.

Engle, M. A., M. S. Gustin, and H. Zhang. 2001. Quantifying natural source mercury emissions from the Ivanhoe Mining District, north-central Nevada, USA. Atmospheric Environment **35**:3987-3997.

Ethier, A., D. Mackay, L. Toose-Reid, N. O'Driscoll, A. Scheuhammer, and D. Lean. 2008. The development and application of a mass balance model for mercury (total, elemental and methyl) using data from a remote lake (Big Dam West, Nova Scotia, Canada) and the multi-species multiplier method. Applied Geochemistry **23**:467-481.

Feng, X., H. Jiang, G. Qiu, H. Yan, G. Li, and Z. Li. 2009. Mercury mass balance study in Wujiangdu and Dongfeng reservoirs, Guizhou, China. Environmental Pollution **157**:2594-2603.

Gao, N., N. G. Armatas, J. B. Shanley, N. C. Kamman, E. K. Miller, G. J. Keeler, T. Scherbatskoy, T. M. Holsen, T. Young, and L. McIlroy. 2006. Mass balance assessment for mercury in Lake Champlain. Environmental Science & Technology **40**:82-89.

Hines, N. A., and P. L. Brezonik. 2007. Mercury inputs and outputs at a small lake in northern Minnesota. Biogeochemistry **84**:265-284.

Jonsson, S. 2013. Unraveling the importance of solid and adsorbed phase mercury speciation for methylmercury formation, evasion and bioaccumulation. Ph.D. Dissertation. Umeå Universitet.

Kocman, D., and M. Horvat. 2011. Non-point source mercury emission from the Idrija Hg-mine region: GIS mercury emission model. Journal of Environmental Management **92**:2038-2046.

Kronberg, R. M., A. Drott, M. Jiskra, J. G. Wiederhold, E. Bjorn, and U. Skyllberg. 2016. Forest harvest contribution to Boreal freshwater methyl mercury load. Global Biogeochemical Cycles **30**:825-843.

Lawson, N. M., R. P. Mason, and J. M. Laporte. 2001. The fate and transport of mercury, methylmercury, and other trace metals in Chesapeake Bay tributaries. Water Research **35**:501-515.

MacLeod, M., T. E. McKone, and D. Mackay. 2005. Mass Balance for Mercury in the San Francisco Bay Area. Environmental Science & Technology **39**:6721-6729.

Mason, R. P., N. M. Lawson, A. L. Lawrence, J. J. Leaner, J. G. Lee, and G.-R. Sheu. 1999. Mercury in the Chesapeake Bay. Marine Chemistry **65**:77-96.

Mason, R. P., N. M. Lawson, and K. A. Sullivan. 1997. Atmospheric deposition to the Chesapeake Bay watershed - Regional and local sources. Atmospheric Environment **31**:3531-3540.

Mason, R. P., and G. R. Sheu. 2002. Role of the ocean in the global mercury cycle. Global Biogeochemical Cycles **16**:14.

Mazur, M., C. P. J. Mitchell, C. S. Eckley, S. L. Eggert, R. K. Kolka, S. D. Sebestyen, and E. B. Swain. 2014. Gaseous mercury fluxes from forest soils in response to forest harvesting intensity: A field manipulation experiment. Science of the Total Environment **496**:678-687.

Muresan, B., D. Cossa, S. Richard, and B. Burban. 2007. Mercury speciation and exchanges at the air–water interface of a tropical artificial reservoir, French Guiana. Science of the Total Environment **385**:132-145.

Nacht, D. M., M. S. Gustin, M. A. Engle, R. E. Zehner, and A. D. Giglini. 2004. Atmospheric mercury emissions and speciation at the sulphur bank mercury mine superfund site, Northern California. Environmental Science & Technology **38**:1977-1983.

Naik, A. P., and C. R. Hammerschmidt. 2011. Mercury and trace metal partitioning and fluxes in suburban Southwest Ohio watersheds. Water Research **45**:5151-5160.

Porvari, P., M. Verta, J. Munthe, and M. Haapanen. 2003. Forestry practices increase mercury and methyl mercury output from boreal forest catchments. Environmental Science & Technology **37**:2389-2393.

Qureshi, A., M. MacLeod, M. Scheringer, and K. Hungerbühler. 2009. Mercury cycling and species mass balances in four North American lakes. Environmental Pollution **157**:452-462.

Rajar, R., M. Četina, M. Horvat, and D. Žagar. 2007. Mass balance of mercury in the Mediterranean Sea. Marine Chemistry **107**:89-102.

Rolfhus, K. R., and W. F. Fitzgerald. 1995. Linkages between atmospheric mercury deposition and the methylmercury content of marine fish. Water Air and Soil Pollution **80**:291-297.

Sakata, M., K. Marumoto, M. Narukawa, and K. Asakura. 2006. Mass balance and sources of mercury in Tokyo Bay. Journal of Oceanography **62**:767-775.

Schartup, A. T., P. H. Balcom, A. L. Soerensen, K. J. Gosnell, R. S. D. Calder, R. P. Mason, and E. M. Sunderland. 2015. Freshwater discharges drive high levels of methylmercury in Arctic marine biota. Proceedings of the National Academy of Sciences **112**:11789-11794.

Shanley, J. B., M. A. Mast, D. H. Campbell, G. R. Aiken, D. P. Krabbenhoft, R. J. Hunt, J. F. Walker, P. F. Schuster, A. Chalmers, B. T. Aulenbach, N. E. Peters, M. Marvin-DiPasquale, D. W. Clow, and M. M. Shafer. 2008. Comparison of total mercury and methylmercury

cycling at five sites using the small watershed approach. Environmental Pollution **154**:143-154.

Soerensen, A. L., D. J. Jacob, A. Schartup, J. A. Fisher, I. Lehnherr, V. L. St Louis, L. E. Heimbürger, J. E. Sonke, D. P. Krabbenhoft, and E. M. Sunderland. 2016. A mass budget for mercury and methylmercury in the Arctic Ocean. Global Biogeochemical Cycles.

Sorensen, R., M. Meili, L. Lambertsson, C. von Bromssen, and K. Bishop. 2009. The Effects of Forest Harvest Operations on Mercury and Methylmercury in Two Boreal Streams: Relatively Small Changes in the First Two Years prior to Site Preparation. Ambio **38**:364-372.

Suchanek, T. H., J. Cooke, K. Keller, S. Jorgensen, P. J. Richerson, C. A. Eagles-Smith, E. J. Harner, and D. P. Adam. 2009. A mass balance mercury budget for a mine-dominated lake: Clear Lake, California. Water, air, and soil pollution **196**:51-73.

Watras, C., K. Morrison, and R. Back. 1996. Mass balance studies of mercury and methyl mercury in small temperate/boreal lakes of the northern hemisphere. Pages 329-358 Global and regional mercury cycles: Sources, fluxes and mass balances. Springer.
